# Low-cost grain sorting technologies to reduce mycotoxin contamination in maize and groundnut

**DOI:** 10.1016/j.foodcont.2020.107363

**Published:** 2020-12

**Authors:** Meriem Aoun, William Stafstrom, Paige Priest, John Fuchs, Gary L. Windham, W. Paul Williams, Rebecca J. Nelson

**Affiliations:** a School of Integrative Plant Science, Cornell University, Ithaca, NY, 14853, USA; b Masters of Public Health Program, Cornell University, Ithaca, NY, 14853, USA; c The Widget Factory, Ithaca, NY, 14850, USA; d USDA, Agricultural Research Service, Corn Host Plant Resistance Research Unit, Mississippi State, MS, 39762, USA

**Keywords:** Aflatoxins, Fumonisins, Food safety, Grain sorting, Maize, Groundnut

## Abstract

The widespread contamination of foods by mycotoxins continues to be a public health hazard in sub-Saharan Africa, with maize and groundnut being major sources of contamination. This study was undertaken to assess the hypothesis that grain sorting can be used to reduce mycotoxin contamination in grain lots by removing toxic kernels. We tested a set of sorting principles and methods for reducing mycotoxin levels in maize and groundnut from a variety of genotypes and environments. We found that kernel bulk density (KBD) and 100-kernel weight (HKW) were associated with the levels of aflatoxins (AF) and fumonisins (FUM) in maize grain. A low-cost sorter prototype (the ‘DropSort’ device) that separated maize grain based on KBD and HKW was more effective in reducing FUM than AF. We then evaluated the effectiveness of DropSorting when combined with either size or visual sorting. Size sorting followed by DropSorting was the fastest method for reducing FUM to under 2 ppm, but was not effective in reducing AF levels in maize grain to under 20 ppb, especially for heavily AF-contaminated grain. Analysis of individual kernels showed that high -AF maize kernels had lower weight, volume, density, length, and width and higher sphericity than those with low AF. Single kernel weight was the most significant predictor of AF concentration. The DropSort excluded kernels with lower single kernel weight, volume, width, depth, and sphericity. We also found that visual sorting and bright greenish-yellow fluorescence sorting of maize single kernels were successful in separating kernels based on AF levels. For groundnut, the DropSort grouped grain based on HKW and did not significantly reduce AF concentrations, whereas size sorting and visual sorting were much more effective.

## Highlights

• The DropSort prototype was more effective in reducing fumonisins than aflatoxins.
• DropSorting following size sorting was effective in reducing fumonisins below 2 ppm.
• Visual sorting was more effective in reducing fumonisins than aflatoxins in maize.
• Size and visual sorting separated groundnut grain based on aflatoxin levels.
• Single kernel mass is the most significant determinant of AF levels.

## 1 Introduction

Efforts to grow and purchase safe food are often hindered by the accumulation of fungal toxins when crops are grown under stress and/or stored under sub-optimal conditions. The UN's Food and Agriculture Organization estimated that 25% of harvested crops are contaminated with mycotoxins (FAO, 2004). This was recently validated, with the further observation that 60–80% of foods carry detectable levels of mycotoxins (Eskola et al., 2019). Over 5 billion people, mostly in developing world, are chronically exposed to aflatoxins (AF; Strosnider et al., 2006, Wild & Gong, 2009), a group of mycotoxins produced by *Aspergillus flavus* and *A. parasiticus*. Acute AF exposure can cause fatal aflatoxicosis (Azziz-Baumgartner et al., 2005; Kamala et al., 2018; Ngindu et al., 1982; Nyikal et al., 2004; Reddy & Raghavender, 2007) whereas chronic exposure is associated with liver cancer, child stunting, and immunosuppression (Gong, Turner, Hall, & Wild, 2008; Kumar, Mahato, Kamle, Mohanta, & Kang, 2017). Fumonisins (FUM), a family of mycotoxins produced by *Fusarium verticillioides* and *F. proliferatum*, contaminate maize (corn) in much of the world. FUM is associated with neural tube defects, child stunting, and esophageal cancer (Shirima et al., 2014, Stockmann-Juvala & Savolainen, 2008).

The regulatory limits for AF vary among countries and intended use. For instance, the AF regulatory limit in food is 4 μg/kg (parts per billion or ppb) in the European Union, 10 ppb in Tanzania, and 20 ppb in the USA (European Commission, 2010; Magoke et al., 2019; USFDA, 2000) For FUM, the regulatory limits vary from 2 to 4 mg/kg (parts per million or ppm) in the USA (USFDA, 2001). The regulatory limit by Codex Alimentarius is 4 ppm for fumonisins B_1_ + B_2_ in unprocessed maize and 2 ppm in maize flour (Codex Alimentarius, 1997). In industrialized countries, mycotoxin regulations are strictly enforced, so mycotoxin contamination is an economic burden on farmers (Mitchell, Bowers, Hurburgh, & Wu, 2016). During mycotoxin-outbreak years in the USA, contaminated grain lots are rejected, forcing farmers to dispose of their crop or accept a lower price on feed markets. In developing countries and especially in rural subsistence farming communities, mycotoxin problems are rarely regulated, so it can represent a serious public health hazard (Zain, 2011).

Maize and groundnut (peanut) are central to human diets in much of sub-Saharan Africa, and are particularly vulnerable to colonization by *A. flavus* and thus to AF accumulation. Maize is also highly susceptible to infection by toxigenic *F. verticillioides* and is frequently contaminated with FUM. Maize and groundnut are therefore the main contributors of mycotoxin exposure in African diet (Shephard, 2008; Wagacha & Muthomi, 2008). The majority of sub-Saharan African maize and groundnut consumers are not protected by regulatory measures that ensure food safety (Strosnider et al., 2006; Zain, 2011). Pre-harvest colonization of maize and groundnut by *A. flavus* and *F. verticillioides* are favored by the plant stresses (Cao et al., 2013; Cotty & Jaime-Garcia, 2007) that are prevalent in tropical regions such as sub-Saharan Africa and the southern States of the USA. Post-harvest toxin accumulation is favored by moist conditions and pest damage, which are characteristic of the inadequate drying and storage conditions that are prevalent in low-resource settings.

Successful mycotoxin management requires strategies implemented both before and after harvest. Pre-harvest management strategies include breeding for host resistance, agronomic practices that reduce crop stress, chemical control, and biological control. Breeding for mycotoxin resistance has been difficult to implement due to the quantitative nature of resistance and high genotype-by-environment interactions (Lanubile et al., 2017; Warburton & Williams, 2014). The plant stress factors that enhance toxin accumulation, such as drought, high temperatures, low soil nitrogen and organic matter, and insect damage, can to some extent be mitigated through crop management practices, though resource-limited smallholder farmers may have limited capacity to implement such practices (Mutiga et al., 2017; Sétamou, Cardwell, Schulthess, & Hell, 1997; Widstrom, McMillian, Beaver, & Wilson, 1990). In sub-Saharan Africa, biological control using native strains of atoxigenic *A. flavus* has led to significant reductions in AF contamination (Bandyopadhyay et al., 2016). Postharvest management strategies include grain sorting, drying, and adequate storage (Murashiki et al., 2018; Waliyar et al., 2014).

In mycotoxin-contaminated grain lots, the distribution of toxin levels among kernels is typically highly heterogeneous (Grenier, Loureiro-Bracarense, Leslie, & Oswald, 2014). Toxic kernels usually occur in low frequency in a grain lot (Stasiewicz et al., 2017; Whitaker & Johansson, 2005), with the majority of the toxin carried by a minority of kernels (Kabak, Dobson, & Var, 2006; Stasiewicz et al., 2017). The heterogeneity of toxin levels means that efforts to quantify toxin levels are subject to high sampling variances. Grain sorting offers an alternative to manage the heterogeneity of toxin levels and can be used to rehabilitate grain lots by sorting out toxic kernels prior to consumption.

For sorting to be effective, the subset of kernels that is most toxic must be distinguishable based on detectable characteristics. Physical attributes such as kernel density, size or color could be indicators of the toxin levels in the grain and therefore might be used to sort grain. A recent study by Morales et al. (2018) showed a negative correlation between kernel bulk density (KBD) and FUM levels in maize kernels. Davidson, Holaday, and Bennett (1981) also showed that excluding the least dense kernels using a gravity table reduced AF contamination in groundnut. Likewise, Whitaker at al. (2005) reported that smaller groundnut kernels tended to have higher concentrations of AF levels than larger kernels. In addition, exclusion of insect-damaged, broken, misshapen, and discolored kernels can help reduce mycotoxin contamination (Afolabi, Bandyopadhyay, Leslie, & Ekpo, 2006; Kabak et al., 2006; Mutiga et al., 2014). Optical methods have also been used to identify potentially toxic kernels. For example, the bright greenish-yellow fluorescence (BGYF) test has been used to identify AF-contaminated grain. Kernels infected with *A. flavus* usually fluoresce under 365 nm ultraviolet (UV) light. This method is not always reliable because other compounds produced by *A. flavus,* such as kojic acid, can also fluoresce under UV light (Dickens and Whitaker, 1981, Schmitt & Hurburgh, 1989; Shotwell & Hesseltine, 1981), even in the absence of AF. Commercial grain sorting based on the kernel attributes described above exist but are too expensive to be practical in low-resource environments.

Low-cost grain sorters effective in reducing mycotoxin contamination are urgently needed to tackle this human and livestock health hazard. The effectiveness of the sorting methods discussed above vary among studies, and it is not clear whether this is due to differences among grain samples, sorting methods, or other factors. The present study was undertaken as part of a larger effort to provide integrated mycotoxin management options, with a focus on approaches that might be relevant in low-resource settings. The potential users and applications of grain sorting technologies could include: 1) consumers in local hammer mills for reducing exposure of low-income communities, such as those in sub-Saharan Africa, 2) grain buyers for reducing sampling variance to improve the estimation of mycotoxin levels in grain lots and 3) breeders for better phenotyping of mycotoxin resistance.

We previously developed a low-cost grain sorter prototype, designated the “DropSort” (Nelson, 2016). The prototype was intended to sort grain based on maize kernel density and has been found to reduce FUM levels in US and Kenyan maize (Stafstrom et al., in prep, Ngure et al., in prep.). The objectives of this study were (1) to test the effectiveness of the DropSort in reducing mycotoxins in maize and groundnut in USA and Tanzania, (2) to test the effectiveness of various sorting methods such as combining DropSorting with size sorting and DropSorting with visual sorting in reducing mycotoxins, and (3) to determine bulk and single kernel attributes associated with toxin levels in maize and groundnut to improve sorting efficiency of future devices.

## 2 Materials and methods

### 2.1 Maize samples

The maize samples used in this study came from experimental plots in the USA and from open markets in Tanzania. For the USA samples, maize hybrids were grown in single row plots in a randomized complete block design (RCBD) in three replicates at the Mississippi State University research farm in Starkville, Mississippi (Tables S1 and S2). Nineteen hybrids were grown in 2017 (MS17), and another seven hybrids were grown in 2018 (MS18). Primary ears were inoculated using the side-needle technique with the highly toxigenic *Aspergillus flavus* strain NRRL 33574.26 (Windham & Williams, 1999). Fungal inoculum preparation and ear inoculation were as described by Warburton et al. (2009). Ears were inoculated seven days after most of the plants per plot had reached silking. Ears were harvested at maturity, approximately 60 days after silking. The inoculated primary ears were harvested, dried, and shelled and samples of ~600 g from each plot were used for analysis. Three plots from three independent hybrids had poor germination in 2017, so kernel samples from 54 plots (out of 57 plots) were used for subsequent analysis. For MS18, samples were prepared from 20 plots (Tables S1 and S2).

Samples from Tanzania were collected in 2018 and 2019 (TZ18 and TZ19; Tables S3 and S4). In 2018, 10 maize grain samples (2 kg/sample) were purchased from 10 small-scale grain merchants located close the Kibaigwa grain market. According to the sellers, the grain lots came from different parts of the Dodoma region, particularly Kongwa district and Dodoma Rural district. The TZ18 grain had been harvested in May of 2018. In 2019, 36 maize samples (1 kg/sample) were randomly collected from markets in Kongwa district (30 samples; 16 samples had been harvested in 2019 and 14 samples had been harvested in 2018) and in Arusha (six samples; two samples harvested in 2019 and four samples harvested in 2018; Table S4).

### 2.2 Groundnut samples

A total of 28 groundnut kernel samples (1 kg/sample) were randomly purchased from small-scale sellers in Kongwa district (19 samples; 17 samples harvested in 2019 and two samples harvested in 2018) and in the Arusha market (nine samples, eight samples harvested in 2019 and a single sample harvested in 2018). While they were purchased in Arusha, some of the groundnut samples originated from other locations (two each were from Arusha and Dodoma; a single sample was from Tabora, Tanzania; and four samples were from Malawi; Table S4).

### 2.3 Aflatoxin quantification

For the inoculated MS17 and MS18 maize kernel samples, 50–70 g/sample were ground to a fine powder with an IKA Tube Mill (IKA® Works, Inc, Wilmington, NC) at Cornell University. From each ground sample, a 9.0-g sub-sample was transferred to a 50-mL centrifuge tube. For aflatoxin quantification in single kernels of MS17, each kernel was placed in a grinding vial and milled to a fine powder by steel bead-beating in a SPEX Geno/Grinder 2000 Sample Prep Shaker Tissue Homogenizer (New Life Scientific, Inc., Cridersville, OH, USA). The sample particle size was about the size of finely-ground coffee particles). The powder from each milled kernel was then transferred to a 2 mL Eppendorf tube and the mass of each sample was measured.

The TZ18 and TZ19 maize and groundnut kernel samples (using 40–70 g each) were ground using a Laboratory blender (Waring® Commercial, Stamford, CT) at the Nelson Mandela African Institute of Science and Technology (NM-AIST) in Arusha. A 9.0-g sub-sample from each ground sample was transferred to a 50-mL centrifuge tube. Single kernel analysis was not performed on Tanzanian samples, because single kernel grinding equipment was not available at the NM-AIST. Thus, small sets of 5 kernels were analyzed. The kernel sets were ground using a Waring CAC103 Grinding Bowl for WSG30 Waring Commercial Spice Grinder (Waring® Commercial, Stamford, CT).

To extract AF, a 5-fold volume of 70% methanol based on sample weight was added to each tube. The centrifuge tubes were then vortexed for approximately 5 min and allowed to settle for 5 min. Thereafter, 1.5 mL of supernatant from each sample was transferred to a 2.0-mL Eppendorf tube and centrifuged at 14,000 rpm for 10 min, and 1 mL of supernatant was then transferred to a new 1.5-mL Eppendorf tube. AF was quantified using Total AF ELISA (Enzyme-Linked Immunosorbent Assay) kits (Helica Biosystems Inc., Santa Ana, CA) as described by the manufacturer. The cross-reactivity of the antibody in the assay was stated by the supplier as 100% for AF B1, 77% for AF B2, 64% for AF G1, and 25% for AF G2. The ELISA standards, Neogen's mycotoxin reference materials AF-corn medium (Neogen Food Safety, MI, USA) and the samples were placed in duplicates in the ELISA plate. The optical density (OD, absorbance at 450 nm) of each well in the ELISA plate was read using a BioTek Synergy 2 multi-mode plate reader paired with Gen5™ software (both from BioTek Instruments, Inc., Winooski, VT). A standard curve was constructed based on OD values, expressed as a percentage, with reference to the AF standards. The concentration of AF in the samples were then measured by interpolation from the standard curve. The AF data quality was checked using an intra-plate coefficient of variation (CV) cutoff of 10% and an inter-plate CV cutoff of 15%. The ELISA range of detection is 0–20 ppb. Samples with AF estimates above 20 ppb were diluted in 70% methanol and re-measured.

### 2.4 Fumonisin quantification

FUM levels were only determined for maize samples from Tanzania (TZ18 and TZ19). The maize samples were ground as described above in subsection 2.3 and FUM quantification was performed as described by Morales et al. (2018). In brief, a 15-g sub-sample from each ground bulk sample was transferred to a 50-mL centrifuge tube. To extract FUM, 30 mL of 90% methanol was added to each 50-mL tube, resulting in a two-fold dilution. The tubes were vortexed for approximately 5 min and then allowed to settle for 5 min before transferring 1.5 mL of supernatant from each sample to a 2.0-mL Eppendorf tube. The supernatant was centrifuged for 10 min, and 1.0 mL of the cleared supernatant was transferred to a new 1.5-mL Eppendorf tube. The resulting sample extract was then diluted 1:20 in distilled H_2_O. FUM was quantified using Total FUM ELISA kits (Helica Biosystems Inc., Santa Ana, CA) as described by the manufacturer. The samples were placed in duplicates in the ELISA plate and a control sample (a FUM-contaminated maize sample from experiment TZ18/TZ19) was used as a check in all ELISA plates. The OD, sample FUM concentrations, and data quality check were as described above for AF quantification. Samples with estimated FUM levels above 2 ppm (ELISA range of detection is 0–2 ppm) were diluted in distilled H_2_O and re-measured.

### 2.5 Size sorting

We explored the effect of size sorting on AF levels using five MS18 samples (~600 g/sample) acquired from two susceptible hybrids (two samples per hybrid) and a resistant hybrid (a single sample) (Table 1 and Table S2). Each of the TZ19 maize and groundnut samples was split into two subsamples. The first subsample of ~600 g was size-sorted and afterwards DropSorted (Fig. S1). The remaining 400-g subsample was DropSorted only (Table 1, Tables S5 and S6). The sorting procedure of samples from TZ19 is described in Fig. S1. Size sorting was performed using a sieve (FLAMAN Grain Cleaning & Handling Systems, Saskatoon, Canada). The sieves used were round-hole screens with different perforation sizes, which allowed us to adjust the size cut-off based on the kernel size of each sample.

**Table 1:** Sample origin, grain sorting treatments, and studied mycotoxins in the maize and groundnut bulk samples, single kernels, and small kernel sets.

Crop	Sample set a	Type of infection	Sorting treatment		Considered mycotoxins
			For bulk analyses	For analyses of single kernels or small sets of kernels	
Maize	MS17	Inoculated plots	DropSorting	DropSorting/visual sorting/BGYF sorting	Aflatoxins
	MS18	Inoculated plots	DropSorting/size sorting b	–	Aflatoxins
	TZ18	Natural infection/open markets	DropSorting + visual sorting	–	Fumonisins
	TZ19	Natural infection/open markets	DropSorting + size sorting	DropSorting + size sorting/visual sorting	Aflatoxins + Fumonisins c
Groundnut	TZ19	Natural infection/open markets	DropSorting + size sorting	DropSorting + size sorting/visual sorting	Aflatoxins

a The samples originated from Mississippi experimental plots in the year of 2017 (MS17), Mississippi experimental plots in the year of 2018 (MS18), open markets in Tanzania in the year of 2018 (TZ18), and open markets in Tanzania in the year of 2019 (TZ19).

b A total of 10 maize samples from MS18 were DropSorted and five samples were size sorted.

c Bulk samples used for fumonisin studies mostly differed from those used for aflatoxin studies. Fumonisins were only analyzed for bulk samples, not for small sets of kernels.

### 2.6 DropSorting

The DropSort device (Fig. S2) was used to separate grain into two fractions. With the DropSort device, grain is fed into a chamber and the heavier falls into the “accept” bin, while a fan blows the lighter kernels into the “reject” bin. The proportion of accepted to rejected kernels was determined by the user based on adjustment of a divider that cuts the stream of grain at a desired setting. To improve the efficacy of sorting and increase the chance of discarding lighter kernels, the grain was sorted three times, only re-sorting the accepted fraction after each of the first two passes. The intent was to separate by density, though differences in grain size and shape can influence sorting efficiency. The DropSort device had been calibrated using 3D printed plastic kernels with different shapes, sizes, and densities (Stafstrom et al., in prep).

DropSorting was conducted by passing maize and groundnut kernels of MS17, MS18, TZ18, and TZ19 samples through the DropSort device (Table 1). For the MS17 and MS18 maize samples, approximately 600 g/sample were DropSorted (Table S1). The maize samples from TZ18 were divided into three subsamples. The first subsample was DropSorted, the second subsample was visually sorted, and the third subsample was DropSorted and then visually sorted (Table S3). The remaining subsample from each of the TZ19 maize and groundnut sample (400 g that was not size sorted) was DropSorted. In addition, the size sorted maize and groundnut samples from TZ19 were then DropSorted (Fig. S1), resulting in four classes of kernels: large and heavy (LH), large and light (LL), small and heavy (SH), and small and light (SL) (Tables S5 and S6).

### 2.7 Manual visual sorting

Manual visual sorting was performed for 208 kernels selected from DropSorted maize samples from MS17, for which single kernel attributes were measured. The kernels were obtained from three hybrids with the lowest AF levels, three hybrids with intermediate AF levels, and three hybrids with the highest AF levels. All nine hybrids had three replicates, except one, which had only two replicates (Table 2). From each of the accepted and rejected DropSort fractions, four kernels per sample were randomly obtained, for a total of 208 kernels (Table S7). The same set of kernels were also visually scored as healthy-looking, moderately defective (slight shape deformation), or unhealthy-looking (Table S7).

**Table 2:** Aflatoxin (AF) concentrations in samples from nine hybrids inoculated with *A. flavus* in a 2017 Mississippi field trial from which single kernels were obtained.

Hybrids	AF replicate 1 (ppb)	AF replicate 2 (ppb)	AF replicate 3 (ppb)	AF mean (ppb)
Mp494 × Mp719	1	1.2	41	14.4
TZAR106 × T173	100	30	–	65
Mp494 x Mp717	150	0.1	16	55
GEMS-0027 × Tx779	33	200	52	95
BH8900VIP3111 (2017)	190	120	120	143
Mp494 × PHW79	690	240	39	323
Pioneer 1745R	900	680	660	747
DK64-69	590	450	2160	1067
Pioneer 2088R	1160	1160	1680	1333

A bulk subsample from each TZ18 maize sample was hand sorted to remove discolored, shriveled, misshapen and damaged kernels (Table 1). This visually-based hand sorting was also performed on the DropSorted maize kernels from TZ18. For this double-sorted fraction, discolored or damaged kernels from the heavy (accepted) fraction of the DropSort were added to the light (rejected) fraction. There was no transfer of kernels from the rejected fraction to the accepted fraction of the DropSort (Table S3).

Visual sorting was further performed on small sets of maize and groundnut kernels from TZ19. Sets of maize kernels from Kongwa (166 sets, 5 kernels/set) were selected from DropSorted samples (heavy, light, LH, LL, SH, and SL kernels) and classified visually into healthy-looking kernels (n = 83 sets) and unhealthy-looking kernels (n = 83 sets, Table S8). Similarly, groundnut kernel sets (106 sets, 5 kernels/set) selected from size-sorted and DropSorted samples from Kongwa and Arusha were classified based on their visual appearance into healthy-looking kernels (n = 53 sets) and unhealthy-looking kernels (n = 53 sets, Table S9).

### 2.8 BGYF sorting

The BGYF test was performed on the selected 208 single kernels of MS17 as follows. The kernels were visualized on the germ and the endosperm sides under UV light (excitation at 365 nm) with a Dino-Lite (Edge AM5216ZT 20x~220x Polarizing VGA 60 FPS Handheld Digital Microscope, Microscope LLC, VA, USA). Based on the visual inspection of the Dino-Lite image, kernels were classified as BGYF or non- BGYF.

### 2.9 Measurement of physical bulk kernel attributes

Before and after each sorting method applied to the bulk (sub)samples, we measured kernel bulk density or test weight (KBD), 100-kernel weight (HKW), toxin concentration, and percentage of rejected kernels in each maize and groundnut sample (Tables S1, S2, S3, S5, and S6). For the unsorted maize samples from MS17, we did not measure HKW. We obtained the mass of 250-mL volume of kernels and the KBD was calculated as the mass of the kernels divided by 250 mL. The HKW was measured by recording the mass of 100 randomly-selected kernels.

### 2.10 Measurements of physical single kernel attributes

Single kernel attributes were determined for the 208 kernels selected from DropSorted maize samples from MS17 described above (Subsection 2.7; see also Table 2).

For each kernel, the length, width, and depth were measured using a digital caliper. Sphericity (roundness) of each kernel was then calculated using the following formula:

$$\text{Sphericity}=\frac{\sqrt[{3}]{\left(\frac{\text{length*width*depth}}{2}\right)}}{\frac{\text{length}}{2}}$$

Single kernel volume was obtained using a ‘VolumeSpinner’ setup, which took 36 digital images of each kernel while it was rotated in front of a SLR camera, with 10° of rotation between images. The ‘volume carving’ algorithm (Roussel, Geiger, Fischbach, Jahnke, & Scharr, 2016) allowed the 3D reconstruction of the kernel and calculation of volume. Single kernel mass was measured on a weighing scale (Intelligent Laboratory Classic Top Loading Balance, 100 g × 0.001 g), after which the density of each kernel was calculated.

### 2.11 Evaluation of the accuracy of grain sorting methods

The effectiveness of DropSorting, BGYF test, and visual sorting in rejecting AF contaminated kernels (>20 ppb) and accepting healthy kernels (≤20 ppb) was assessed using the 208 kernels from MS17. Similarly, the accuracy of visual sorting of maize and groundnut kernel sets from TZ19 was assessed based on an AF cutoff of 20 ppb. We calculated sensitivity, specificity, false positive rate (FPR), and false negative rate (FNR) of the each sorting method. Sensitivity (1−FNR) was defined as the frequency of true positives or the number of kernels correctly classified as contaminated, divided by the total number of contaminated kernels. Specificity (1−FPR) was defined as the frequency of true negative or the number of kernels correctly classified as healthy divided by the total number of healthy kernels.

### 2.12 Statistical analysis

Because the toxin data were not normally distributed, AF and FUM data were natural-log (Ln) transformed before statistical analysis. To account for the samples with AF = 0 ppb, 1 ppb was added to AF measures of all the samples. Similarly, for FUM, 0.1 ppm was added to FUM measures of all the samples. The correlations between bulk and single kernel attributes and toxin levels were evaluated using pairwise Pearson's correlations. Tukey's Honest Significant Difference test was used to compare between the effects of each sorting method on bulk and single kernel attributes. For pairwise comparisons, we used the two-tailed *t*-test. Data analysis was performed using R 3.4.1 (R Core Team 2017).

## 3 Results

### 3.1 AF and FUM levels in maize samples

AF concentrations in the MS17 and MS18 samples ranged from 0 to 3536 ppb, with an average of 340 ppb (Fig. S3). Only 12 samples (plots) of the 74 plots (16%) corresponding to the resistant hybrids (Mp494 × Mp717, Mp717 × Mp719, Mp494 × Mp719, Mp715 × Mp717, Mp10:135 × PHW79, Mp313E × Va35, and Mp313E × Mo18W) had AF levels ≤ U.S. Food and Drug Administration (FDA) regulatory limit of 20 ppb. All the remaining samples had AF levels above 20 ppb (Tables S1 and S2).

AF levels in TZ18 samples were low, ranging from 0.3 to 1.7 ppb (Table S3). The AF concentrations in the TZ19 samples were up to four orders of magnitude higher, ranging from 0.7 to 17572 ppb, with an average of 2816 ppb. For TZ19, 23 samples (64%) had AF above 20 ppb, 21 samples (58%) had AF levels of greater than 100 ppb, and 14 samples (39%) had AF exceeding 1000 ppb (Fig. 3, Table S4). The samples collected from the Kongwa district had higher AF concentrations (n = 30, mean = 3291 ppb) than those collected from Arusha markets (n = 6, AF mean = 395 ppb). Of the 23 samples with AF above 20 ppb, five samples were collected from Arusha and 18 samples were obtained from Kongwa (Table S4).

**Fig. 1:**
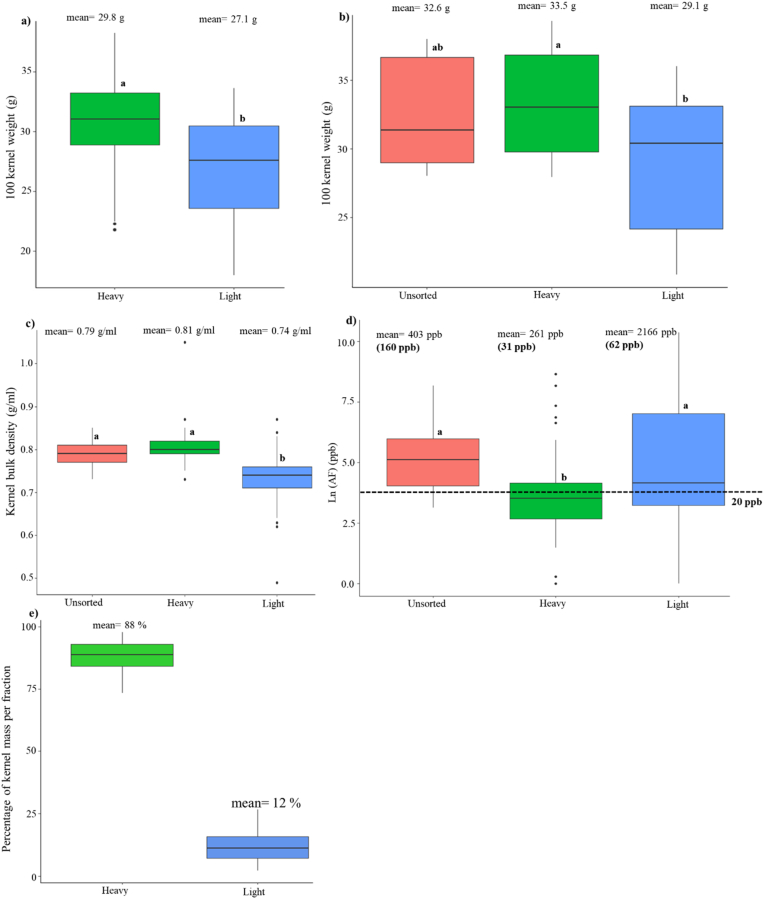
DropSorting effects on bulk kernel attributes in maize grain from experimental plots in Mississippi. Kernel samples were from inoculated ears with *Aspergillus flavus* in field trials in Starkville, Mississippi (MS) in the years of 2017 (48 samples, 19 hybrids) and 2018 (10 samples, five hybrids). a) DropSorting effects on 100 kernel weight of samples from MS17, b) DropSorting effects of 100 kernel weight of samples from MS18, c) DropSorting effects on kernel bulk density of samples from MS17 & MS18, d) DropSorting effects on Ln (aflatoxins) in parts per billion (ppb) of samples from MS17 & MS18, the aflatoxin mean and median (shown in parentheses) of each fraction are presented on the boxplots, and e) percentage of kernel mass in the DropSorted kernel fractions. The letters on the boxplots represent results of Tukey's test (treatments with different letters are significantly different at 95% level of confidence.

**Fig. 2:**
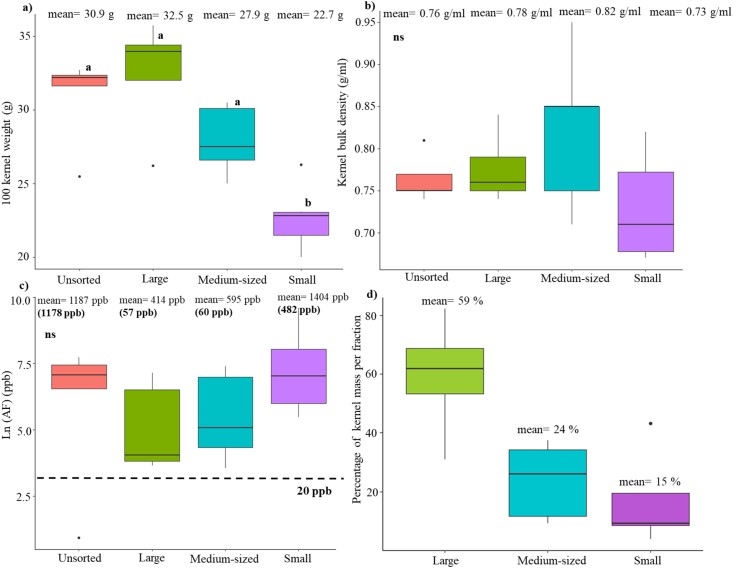
Size sorting effects on the bulk attributes in maize grain from experimental plots in Mississippi. Grain samples (n = 5) were obtained from maize ears inoculated with *Aspergillus flavus* in field trials in Starkville, Mississippi (MS) in 2018. a) Size sorting effects on 100 kernel weight, b) size sorting effects on kernel bulk density c) size sorting effects on Ln (aflatoxins) (in ppb), the aflatoxin mean and median (shown in parentheses) of each fraction are presented on the boxplots, and d) percentage of kernel mass in the size sorted kernel fractions. The letters on the boxplots represent results of Tukey's test (treatments with different letters are significantly different, ns: no significant difference between treatments at 95% level of confidence).

**Fig. 3:**
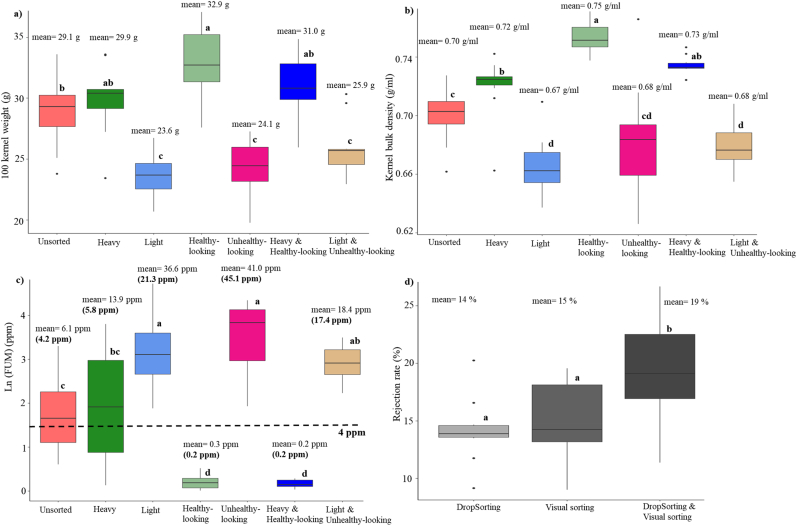
Effects of DropSorting, visual sorting, and DropSorting followed by visual sorting on bulk kernel attributes of maize samples (n = 10) collected from open markets in Tanzania in 2018. a) Sorting method effects of on 100 kernel weight, b) sorting method effects on kernel bulk density, c) sorting method effects on Ln (fumonisins) (ppm), the fumonisin mean and median (shown in parentheses) of each fraction are presented on the boxplots, and d) rejection rates of sorting methods. The letters on the boxplots represent results of Tukey's test (treatments with different letters are significantly different at 95% level of confidence).

FUM concentrations in TZ18 were high, ranging from 0.8 to 26.1 ppm with an average of 6.1 ppm (Fig. 3, Table S3). The FUM concentrations in the 36 samples from TZ19 ranged from 0 to 14.9 ppm, with an average of 2.6 ppm (Fig. S3, Table S4). A total of 26 samples (72%) from TZ19 had detectable FUM, eight of them had FUM levels greater than 4 ppm. Of these eight samples, seven were from Kongwa and a single sample was from Arusha. We found that most of the highly toxic samples had either high AF or high FUM, with the exception of two samples that had extremely high AF (>1000 ppb) and FUM (>14 ppm) (Table S4). The TZ19 maize samples collected in Kongwa and harvested in 2018 had an average AF level of 1886 ppb and FUM level of 3.0 ppm, while those harvested in 2019 had an average AF level of 4521 ppb and FUM level of 2.8 ppm. Four of the samples collected in Arusha markets and harvested in 2018 had an average AF level of 651 ppb and FUM level of 0.2 ppm, while the remaining two Arusha samples harvested in 2019 had an average AF level of 44 ppb and average FUM level of 4.8 ppm (Table S4).

### 3.2 DropSorting effects on AF levels and physical bulk kernel attributes in maize grain inoculated with *A. flavus* in Mississippi

Of the 69 MS17 and MS18 maize samples, 58 (48 from MS17 and 10 from MS18) had AF levels over 20 ppb (the U.S. FDA legal limit in food); these were sorted using the DropSort device. The DropSort average rejection rate was 12%. The accepted (heavy) kernels had significantly higher HKW and KBD compared to the rejected (light) kernels, while no significant difference was observed between the unsorted and the DropSort accepted kernels in terms of HKW and KBD. For the DropSorted MS17 and MS18 samples, the average KBD of the unsorted, accepted and rejected fractions was 0.79, 0.81, and 0.74, respectively. For the samples from MS18, the mean of HKW in the unsorted, accepted and rejected fractions was 32.6, 33.5, and 29.1 g/mL, respectively. Similarly, in MS17 samples, HKW was significantly higher in the DropSort accepted kernels compared to that in the rejected kernels (Fig. 1, Table S1).

The DropSorting significantly reduced AF levels in the samples procured from MS. The average AF level in the rejected kernels of the DropSort was 8-fold higher than that in the accepted kernels. The mean of AF in accepted kernels of the sorter was 261 ppb (median = 31 ppb), which represented 65% of the average AF level in the unsorted grain. There was no significant difference between AF levels in the unsorted kernels (mean = 403 ppb) and in the rejected kernels (mean = 2166 ppb) (Fig. 1, Table S1).

### 3.3 Size sorting effects on AF levels and physical bulk kernel attributes in maize grain inoculated with *A. flavus* in Mississippi

The size-sorted kernels from the five MS18 samples were separated into three classes: large, medium and small. On average, the percentage of large, medium, and small kernels were 59%, 24% and 15%, respectively (Fig. 2). We used grain screens with perforation diameters of 0.75 cm and 0.87 cm to create the three kernel size classes, with the exception of a single sample that belonged to the AF resistant hybrid Mp715 × Mp717. For the latter, we used grain screen perforation diameters of 0.75 cm and 0.79 cm because the kernels of this hybrid were smaller.

Size sorting had a significant effect on HKW but no significant effect on KBD. Smaller kernels had significantly lower HKW compared to those of unsorted, large, and medium sized kernels. Size sorting based on this small sample number (n = 5) showed no significant impact on AF levels. Although the difference was not significant, the average AF concentration in large and medium sized kernels was 9-fold and 6-fold lower, respectively, than AF levels in small kernels (Fig. 2, Table S2).

### 3.4 Effects of DropSorting, visual sorting, and a combination thereof on FUM levels and physical bulk kernel attributes in naturally contaminated maize samples from TZ18

DropSorting and visual sorting of TZ18 samples (n = 10) had significant effects on HKW and KBD. Heavy kernels (the DropSort-accepted fraction) and healthy-looking kernels had higher HKW and KBD compared to those in the light kernels (the DropSort-rejected fraction) and the unhealthy-looking kernels. The average rejection rates of visual sorting and DropSorting were approximately 14%, while the average rejection rate for combined DropSorting and visual sorting was 19% (Fig. 3).

Based on an average rejection rate of 14%, the FUM levels in the DropSort heavy fraction were not significantly different compared to that in the unsorted samples. However, the average FUM in the DropSort heavy fraction (FUM mean = 14.7 ppm, median = 5.8 ppm) was lower than in the light fractions (FUM mean = 33.5 ppm, median = 21.3 ppm). Visual sorting outperformed the DropSorting in reducing FUM levels. For instance, FUM level in the healthy-looking kernels was, on average, 0.3 ppm compared to 41.0 ppm in the unhealthy-looking kernels. Double-sorting (DropSorting + visual) reduced the levels of FUM to an average of 0.2 ppm for the heavy + healthy-looking kernels compared to 18.4 ppm for the light + unhealthy-looking kernels (Fig. 3, Table S3). The average sorting speed of the DropSorting, visual sorting, and DropSorting + visual sorting was 44 min/kg, 47 min/kg, and 74 min/kg, respectively (Fig. S4).

### 3.5 Effects of size sorting, DropSorting, and a combination thereof on FUM and AF levels and physical bulk kernel attributes in naturally contaminated maize samples from TZ19

**Fig. 4:**
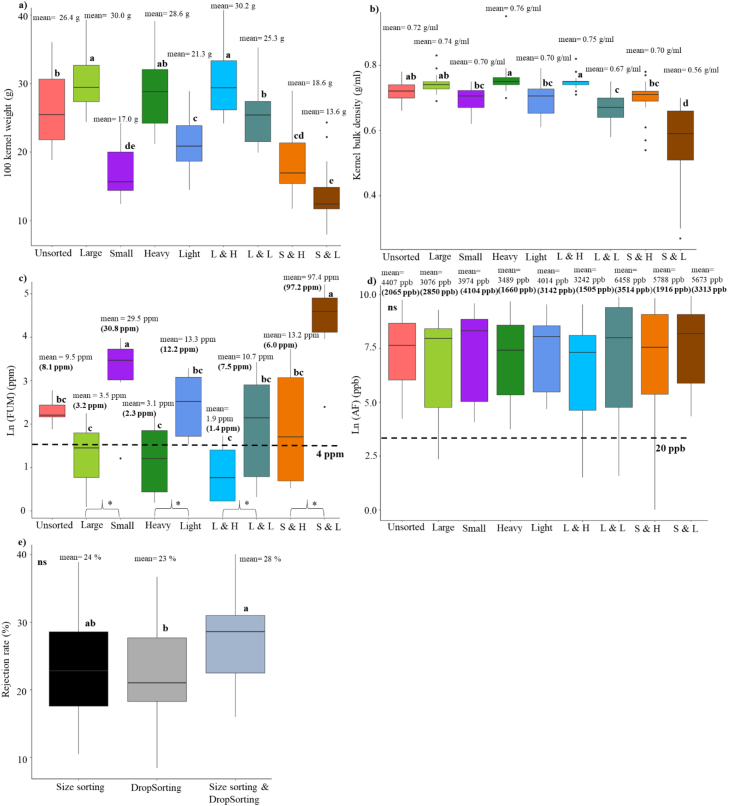
Effects of size sorting, DropSorting, and size sorting followed by DropSorting on bulk kernel attributes of maize samples collected from open markets in Tanzania in 2019. a) Sorting method effects on 100 kernel weight (n = 29 samples), b) sorting method effects on kernel bulk density (n = 29 samples), c) sorting method effects on Ln (fumonisins) in (ppm) (n = 8 samples), the fumonisin mean and median (shown in parentheses) of each fraction are presented on the boxplots, d) sorting method effects on Ln (aflatoxins) in (ppb) (n = 23 samples), the aflatoxin mean and median (shown in parentheses) of each fraction are presented on the boxplots, and e) rejection rates of sorting methods. L & H: large and heavy kernels, L & L: large and light kernels, S & H: small & heavy kernels, and S & L: small and light kernels. The letters on the boxplots represent results of Tukey's test (treatments with different letters are significantly different, ns: no significant difference between treatments at 95% level of confidence). Pairwise comparisons based on two tail *t*-test in Fig. 4c are presented with the symbol * that corresponds to significant difference at 95% level of confidence).

We investigated the effectiveness of combining size sorting and DropSorting in reducing mycotoxin levels in naturally infected maize samples from TZ19. We performed sorting on 23 maize samples with AF levels >20 ppb and eight samples with FUM >4 ppm (Table S5). Only two samples had AF > 20 ppb and FUM >4 ppm. The rejection rate of the DropSort ranged from 8% to 37% with an average of 23% (Fig. 4). Size sorting separated the kernels into large and small kernels using grain screen perforation diameters of 0.75 or 0.79 cm in diameter depending on the kernel size of unsorted samples. The size sorting rejection rate ranged from 10% to 39%, with an average of 24% (Fig. 4). Size sorting was faster, at 5 min/kg on average, compared the DropSort. The rejection rate of the combined size sorting and DropSorting was 28% on average, where the accepted fraction corresponds to large + heavy kernels (Fig. 4).

DropSorting had a significant effect on HKW and KBD, while size sorting had significant effect on HKW but not on KBD (Fig. 4a and b). As illustrated in Fig. 4c, size sorting and DropSorting significantly reduced FUM levels. For instance, the large kernels (mean = 3.5 ppm) and heavy kernels (mean = 3.1 ppm) had significantly lower FUM levels compared to that of small kernels (mean = 29.5 ppm) and light kernels (mean = 13.3 ppm). The mean FUM levels in the large kernels and the heavy kernels were below the 4 ppm regulatory limit set by Codex Alimentarius for unprocessed maize. Combining both sorting methods showed reduced levels (large + heavy kernels had an average FUM level of 1.9 ppm), while small + light kernels had the highest levels of FUM (mean = 97.4 ppm). Therefore, combining size and DropSorting can reduce FUM levels.

As shown in Fig. 4d, neither size sorting nor DropSorting had a significant impact on AF levels: all the sorting fractions had extremely high AF levels. This is most likely because the TZ19 samples in this study were so highly contaminated with AF that there was no clean fraction available.

### 3.6 Physical bulk kernel attributes associated with AF and FUM levels in maize grain

Pearson correlations of bulk kernel attributes for combined unsorted samples from Mississippi and Tanzania showed negative correlations between KBD and AF levels (r = −0.3, *P* ≤ 0.01) and between HKW and AF concentrations (r = −0.6, *P* ≤ 0.001). On the other hand, there was no significant correlation between FUM levels, KBD, and HKW for the TZ18 and TZ19 unsorted samples most likely because of the small sample size of FUM-contaminated samples in this study (Fig. S5).

To increase the sample size, we studied the correlation between toxin levels and bulk kernel attributes (HKW, KBD) in the combined unsorted and sorted maize samples from each location USA (MS17 and MS18), TZ18, and TZ19. This showed that AF was negatively correlated with KBD (r = −0.28, *P* value ≤ 0.001) in the combined unsorted and sorted MS samples (Fig. S6a). Similarly, there was a significant negative correlation between AF and KBD (r = −0.21, *P* value < 0.01) and between AF and HKW (r = −0.30, *P* value < 0.001) in samples from TZ19 (Fig. S6b). There were stronger negative correlations between FUM and bulk kernel attributes (HKW and KBD) than those observed with AF, explaining the better sorting effectiveness for FUM based on these physical kernel attributes. For instance for the combined unsorted and sorted TZ19 maize, there was a negative correlation between FUM and KBD (r = −0.50, *P* value < 0.001) and between FUM and HKW (r = −0.70, *P* value < 0.001). Similarly, in the maize samples from TZ18, FUM was negatively correlated with KBD (r = −0.74, *P* value < 0.001) and HKW (r = −0.59, *P* value < 0.001, Fig. S6c).

### 3.7 Effects of DropSorting on physical single kernel attributes associated with AF levels in maize grain inoculated with *A. flavus*

We investigated the associations between individual kernel attributes and AF levels to seek grain features that could be used to effectively sort grain to reduce AF. We also studied the effects of DropSorting on single kernel physical attributes. For this work, we randomly selected 208 kernels from each of the DropSorted samples from nine hybrids inoculated with *A. flavus* in MS17 (Table 2, Table S7). A total of 104 kernels were selected from the heavy fraction and 104 kernels were selected from the light fraction. Comparisons based on single kernel attributes showed that single kernel mass, volume, width, depth, and sphericity were significantly higher in heavy faction kernels compared to light faction kernels. Single kernel density, length, and AF concentration were not significantly affected with DropSorting (Table 3). The distributions of these eight kernel attributes in DropSort heavy and light fractions are illustrated in Fig. S7.

**Table 3:** Differences in the mean values of MS17 maize single kernel attributes between the DropSort fractions and aflatoxin levels.

Single kernel attributes	DropSort fractions		Aflatoxin classes	
	Heavy kernels (n = 104)	Light kernels (n = 104)	Low AF (≤20 ppb) (n = 137)	High AF (>20 ppb) (n = 69)
Kernel aflatoxin (ppb)	187 (a)	2724 (a)	–	–
Kernel mass (g)	0.34 (a)	0.28 (b)	0.32 (a)	0.29 (b)
Kernel density (g/cm^3^)	0.96 (a)	0.91 (a)	0.96 (a)	0.90 (b)
Kernel length (mm)	11.6 (a)	11.5 (a)	11.7 (a)	11.2 (b)
Kernel width (mm)	8.7 (a)	8.4 (b)	8.6 (a)	8.4 (b)
Kernel depth (mm)	4.5 (a)	4.1 (b)	4.2 (a)	4.4 (a)
Kernel volume (mm^3^)	361 (a)	313 (b)	345 (a)	321 (b)
Kernel sphericity	0.7 (a)	0.6 (b)	0.64 (a)	0.66 (b)

The letters in parentheses represent results of two-tail *t*-test (treatments with different letters are significantly different at 95% level of confidence).

A total of 137 kernels had AF levels of less than or equal to 20 ppb, 53 kernels had AF between 21 ppb and 100 ppb, and 16 kernels had AF levels of greater than 100 ppb. Kernels with low levels of AF (≤20 ppb) had significantly higher kernel mass, volume, density, length, and width. In addition, kernels with higher sphericity tended to be more toxic (Table 3). Based on an AF cutoff of 20 ppb, the overall accuracy of DropSorting was 54.7% with a specificity of 71.2% and a sensitivity of 38.2%. DropSorting had a high rate of rejecting healthy kernels with AF levels of less than 20 ppb (61.8%) and accepting toxic kernels with AF levels of greater than 20 ppb (28.8%) (Table 4, Table S7).

**Table 4:** Accuracy of visual sorting, DropSorting, and bright green yellow fluorescence (BGYF) sorting based on aflatoxin concentration in maize and groundnut kernels.

Crop	Location/Year	Sorting a	Accuracy (%)^b^	Specificity (%)^b^	Sensitivity (%)^b^
Maize	MS17	DropSort	54.7	71.2	38.2
		Visual	84.6	69.2	100
		BGYF	85.8	71.6	100
	TZ19	Visual	65.9	38.0	93.8
Groundnut	TZ19	Visual	85.3	78.8	91.7

a Sorting was based on single maize kernels from Mississippi in 2017 (MS17). Visual sorting of maize and groundnut was based on kernel sets (5 kernels/set) from Tanzania in 2019 (TZ19).

b Accuracy of sorting was based on aflatoxin cutoff of 20 ppb (U.S. FDA regulatory limit).

There were significant correlations between AF levels and four of the measured single kernel attributes. Single kernel AF level was negatively correlated with kernel weight (r = −0.31, *P* value ≤ 0.001), kernel density (r = −0.29, *P* value ≤ 0.001), kernel length (r = −0.17, *P* value ≤ 0.05), and kernel width (r = −0.19, *P* value = 0.01). Single kernel weight was the most significant predictor of AF concentration in single kernels (Fig. S8).

### 3.8 Visual sorting and BGYF sorting effects on AF levels in maize grain

**Fig. 5:**
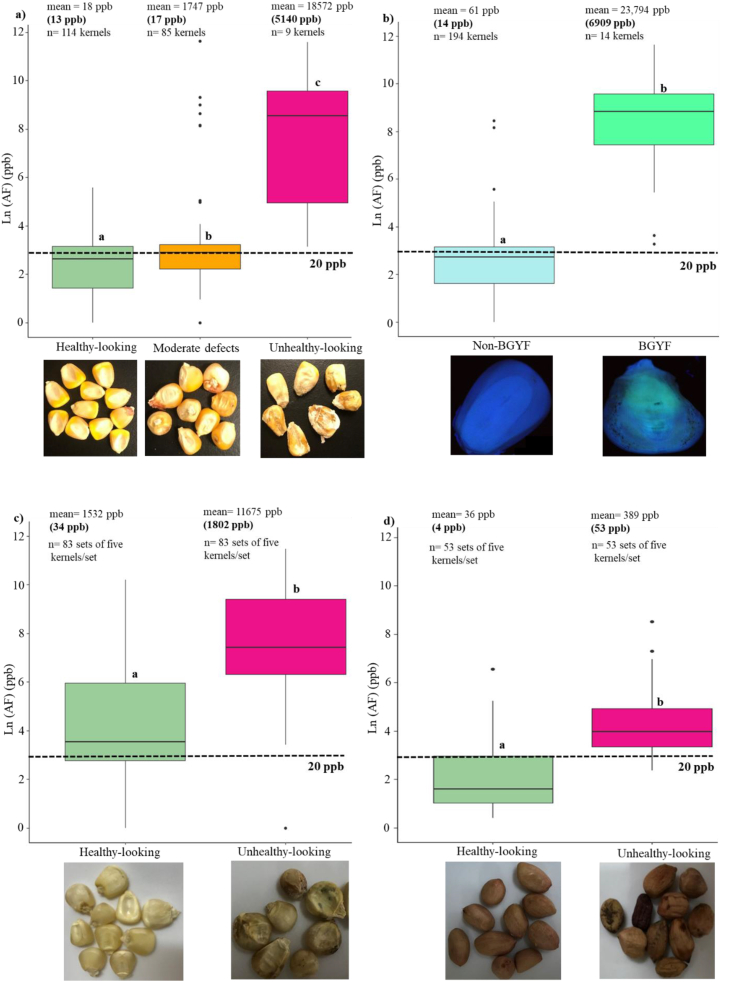
Visual sorting and bright greenish-yellow fluorescence (BGYF) test effects on aflatoxin (AF)concentrations in maize and groundnut kernels. a) Visual sorting effects on AF levels in maize single kernels (n = 208) originated from nine hybrids inoculated with *Aspergillus flavus* in Mississippi field trail in 2017 (MS17), b) BGYF test effects on AF levels in maize single kernels (n = 208) from MS17, c) visual sorting effects on maize kernels (n = 166 kernel sets, five kernels/set) from naturally infected kernels collected from open markets in Tanzania in 2019, and d) visual sorting effects on groundnut kernels (n = 53 kernel sets, five kernels/set) naturally contaminated with AF collected from open markets in Tanzania in 2019. The aflatoxin mean and median (shown in parentheses) of each kernel class are presented on the boxplots The letters on the boxplots represent results of Tukey's test/two tail *t*-test (treatments with different letters are significantly different at 95% level of confidence). (For interpretation of the references to color in this figure legend, the reader is referred to the Web version of this article.)

Based on visual sorting of the 208 kernels selected from the DropSorted MS17 samples, 114 kernels appeared healthy, 85 kernels had moderate defects (slight shape deformation), and nine kernels were unhealthy-looking. The AF levels in these three visually sorted kernel types were significantly different (Fig. 5a). Of these kernels, 48 healthy-looking kernels, 48 kernels with moderate defects, and eight unhealthy-looking kernels were found in the light fraction of the DropSort (Table S7). Based on an AF cutoff of 20 ppb and by grouping healthy-looking kernels and kernels with moderate defects as accepted kernels and unhealthy-looking kernels as rejected kernels, the overall accuracy of visual sorting was 84.6% with a specificity of 69.2% and a sensitivity of 100% (Table 4). The challenge for visual sorting was that 30.8% of kernels with AF levels > 20 ppb were asymptomatic.

The same 208 kernels from MS17 were classified using the BGYF test into 194 non-BGYF kernels and 14 BGYF kernels. Of these BGYF kernels, one had been classified as healthy-looking, seven had moderate defects, and six were unhealthy-looking. The average AF level in non-BGYF kernels and BGYF kernels was 61 ppb (median = 14 ppb) and 23,794 ppb (median = 6909 ppb), respectively; AF levels in the non-BGYF kernels were on average 390-fold lower than in BGYF kernels (Fig. 5b). Thirteen of the 14 BGYF kernels were found in the light fraction of the DropSort (Table S7). Based on an AF cutoff of 20 ppb, the overall accuracy of BGYF test was 85.8% with a specificity of 71.6% and a sensitivity of 100%. The problem with the BGYF test in our study were the 29.4% of kernels with AF levels > 20 ppb that did not fluoresce under UV light (Table 4).

Maize kernels sets (n = 166 sets, five kernels/set) selected from the sorted samples from Kongwa in TZ19 were classified visually into healthy-looking kernels (n = 83 sets) and unhealthy-looking kernels (n = 83 sets, Fig. 5c). The average AF levels in the unhealthy-looking kernels was 11675 ppb (median = 1802 ppb), eight-fold higher than for the healthy-looking kernels (AF mean = 1532 ppb, median = 34 ppb) (Fig. 5c, Table S8). Based on an AF cutoff of 20 ppb, the overall accuracy of visual sorting in naturally infected maize kernels in TZ19 was 65.9%, with a specificity of 38.0% and a sensitivity of 93.8% (Table 4). The limitation for visual sorting were the 62% of asymptomatic toxic kernels with AF levels above 20 ppb. In addition, five kernel sets (6%) were classified as unhealthy-looking but had no detectable AF.

### 3.9 AF levels in groundnut samples from TZ19

All TZ19 groundnut samples had detectable AF except three samples from Arusha grain markets. Fourteen groundnut samples (50% of the samples) had AF levels greater than 20 ppb. Eight of these contaminated samples came from Kongwa and six were from Arusha. Even though the six contaminated samples were procured from Arusha markets, only two samples originated from Arusha while the remaining samples were from elsewhere in Tanzania (one sample from Tabora and two from Dodoma), and one sample was from Malawi. There were samples with extremely high AF levels; nine samples had AF levels greater than 100 ppb and three samples had AF levels greater than 1000 ppb (Fig. S9, Table S4).

### 3.10 Effects of size sorting, DropSorting, and a combination thereof on AF levels and physical bulk kernel attributes in naturally contaminated groundnut samples from TZ19

**Fig. 6:**
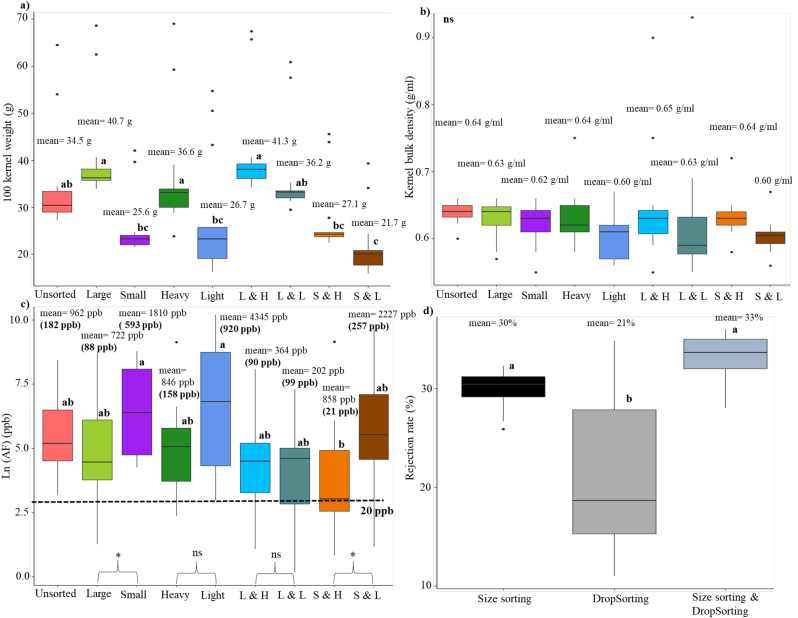
Effects of size sorting, DropSorting, and size sorting followed by DropSorting on bulk kernel attributes of groundnut samples (n = 14) collected from open markets in Tanzania in 2019. a) Sorting method effects on 100 kernel weight, b) sorting method effects on kernel bulk density, c) sorting method effects on Ln (aflatoxins) in (ppb), the aflatoxin mean and median (shown in parentheses) of each fraction are presented on the boxplots, and d) rejection rates of sorting methods. L & H: large and heavy kernels, L & L: large and light kernels, S & H: small & heavy kernels, and S & L: small and light kernels. The letters on the boxplots represent results of Tukey's test (treatments with different letters are significantly different, ns: no significant difference between treatments at 95% level of confidence). Pairwise comparisons based on two tail *t*-test in Fig. 6c are presented with the symbols ns (no significant difference) and * (significant difference at 95% level of confidence).

Fourteen groundnut samples with AF levels of greater than 20 ppb were 1) size sorted, 2) DropSorted, and 3) size and DropSorted. The DropSort rejection rate of groundnut samples ranged from 11.0 to 35%, with an average of 21%. The size sorting rejection rates were much higher, ranging from 26 to 32%, with an average of 30% (Fig. 6). Sorting the groundnut samples with the DropSort was much faster (averaging 14 min/kg, Fig. S4) than for maize samples (mean = 44 min/kg), because the groundnut kernels have a round dimension, allowing them to travel more rapidly through the channel between the sorter's hopper and sorting chamber. The speed of size sorting was faster (averaging 5 min/kg) than DropSorting. The rejection rate of the combined size sorting and DropSorting was 33% on average, where the accepted fraction corresponds to large + heavy kernels (Fig. 6).

As expected, large kernels and heavy kernels had increased HKW compared to the small kernels and the light kernels. Large and heavy (LH) kernels had the highest HKW, while small and light (SL) kernels had the lowest HKW. We found that none of the sorting methods had significant effects on KBD of the groundnut samples (Fig. 6a and b, Table S6). As illustrated in Fig. 6c, small kernels, light kernels, and SL kernels had the highest AF levels. A pairwise comparison between AF levels in large and small kernels showed that small kernels had significantly higher AF levels (mean = 1810 ppb, median = 593 ppb) compared to that in large kernels (mean = 722 ppb, median = 88 ppb). DropSort was not effective in reducing AF levels in groundnut, although SH kernels were less toxic than SL kernels. None of the groundnut sorting classes had AF levels below the U.S. FDA regulatory limit (Fig. 6c).

Among the 28 unsorted groundnut samples, the AF level was not correlated with either KBD or HKW (Fig. S10a). The correlation between AF and bulk kernel attributes in the combined unsorted and sorted groundnut samples (to increase the sample size) showed a negative correlation between AF and HKW (r = −0.34, *P* < 0.001) (Fig. S10b).

### 3.11 Visual sorting effect on AF levels in groundnut kernels

The AF level in unhealthy-looking kernel sets was significantly higher (AF mean = 389 ppb, median = 53 ppb) compared to healthy-looking kernels (AF mean = 36 ppb, median = 4 ppb) (Fig. 5d, Table S9). Visual sorting was much more efficient for sorting groundnut than maize, but was time consuming (mean = 79 min/kg, Fig. S4). Based on an AF cutoff of 20 ppb, the overall accuracy of visual sorting in naturally infected groundnut kernels in TZ19 was 85.3% with a specificity of 78.8% and a sensitivity of 91.7% (Table 4).

## 4 Discussion

With the aim of developing low-cost sorting tools that can be used in low-resource environments under high mycotoxin risk, we explored the effectiveness of a range of approaches to grain sorting of maize and groundnut for reducing mycotoxin levels. Sorting methods included the DropSort device, size sorting, and visual inspection under ambient and UV light. We used grain from diverse environments and genotypes inoculated with toxigenic *A. flavus* and naturally contaminated with mycotoxins. Aflatoxin levels were extremely high in the marketed grain samples of maize and groundnut collected in 2019 from open markets in Tanzania. During the 2019 growing season, Kongwa farmers experienced a drought that led to massive AF contamination. The contamination seen in the TZ18 samples was less severe, but around half of the TZ19 maize samples in our study were harvested in 2018. Therefore, the high AF levels in the TZ19 maize samples that were harvested in 2018 may be due to suboptimal storage conditions, which are known to increase mycotoxin levels (Maina, Wagacha, Mwaura, Muthomi, & Woloshuk, 2016; Pitt, Taniwaki, & Cole, 2013; Walker, Jaime, Kagot, & Probst, 2018).

We found that AF levels were negatively correlated with kernel bulk density (KBD) and 100-kernel weight (HKW) in maize grain. This extends the findings of Morales et al. (2018), who found a significant negative correlation between KBD and FUM levels. These results are consistent with the idea that density and size sorting might be useful for reducing mycotoxin levels. We had devised the low-cost DropSort to sort grain based on maize kernel density with intent to mitigate AF and FUM levels in low-resource settings (Nelson, 2016). Our DropSorting tests using maize samples inoculated with *A. flavus* in Mississippi showed that the device was effective in reducing AF, but not below regulatory limits. In our tests using highly AF-contaminated maize samples from Tanzanian markets, the DropSort was not effective in reducing AF levels.

The DropSort was successful in reducing FUM levels, which agrees with findings of Stafstrom et al. (in prep.) and Ngure et al. (in prep.). The DropSort was more effective in reducing FUM concentrations in TZ19 maize samples compared to TZ18 samples. This is most likely associated with the difference in the DropSort rejection rate, which was 14% for TZ18 samples and 23% for TZ19 samples. For both AF and FUM, toxin concentrations in the light fractions were usually higher than those in the heavy fractions. Therefore, the toxin levels in light fractions could be an indicator of the toxicity level in the unsorted grain lot. Since a grain lot is very heterogeneous with the majority of mycotoxin typically concentrated in a small fraction of kernels (Stasiewicz et al., 2017; Whitaker, Richard, Giesbrecht, Slate, & Ruiz, 2003), the DropSort can reduce sampling variance by creating more homogeneous grain fractions in terms of mass, volume, size (kernel width and depth), shape (roundness) and toxicity. Grain sorting could therefore be useful to grain buyers for improved estimation of mycotoxins in grain lots and to breeders for more effective phenotyping of mycotoxin resistance.

Density sorting methods, including gravity tables, have been used in previous studies with varying results with regard to mycotoxin reduction. For instance, Tkachuk, Dexter, and Tipples (1991) reported that removal of severely infected (thin and shriveled) kernels reduced mycotoxin levels in wheat. Similarly, Shi, Stroshine, & Ileleji (2014) reported that a gravity table was successful in reducing AF levels to below the U.S. FDA regulatory limit in naturally contaminated maize grain. However, Brekke, Peplinski, and Griffin (1975) found that a gravity table was not effective in reducing AF levels in maize. These differences in sorting effectiveness may be due to differences in grain lots -- the inherent properties of the genotypes and the consequences of genotype-by-environment interactions that influence density and toxin amount and distribution -- as well as the sorting technologies and protocols utilized. Furthermore, although gravity tables worked in some cases, they are costly and thus likely inaccessible for rural communities in developing countries.

We found associations among AF levels and maize single kernel characteristics such as mass, density, size, and sphericity. It is logical that fungal colonization influences kernel mass and density, as the process of microbial growth requires consumption of the kernel's resources. The association between toxin and kernel size may suggest that early infection may reduce kernel growth, or that a kernel's position on the maize ear influences its vulnerability to colonization or toxin accumulation. The difference in sphericity between healthy and contaminated kernels could be explained by the fact that natural infection of the ear by *A. flavus* usually occurs near the tip of the ear. However, in the case of inoculated ears, the inoculation method could influence these observations. Further work is required to determine whether infection causes reduced kernel size, or if kernel position and size influences the likelihood of contamination, or if the colonization of entire ears leads to correlated outcomes with regard to kernel traits and toxin levels. The DropSort was successful in sorting based on single kernel mass, volume, width, depth but not based on single kernel density or length. In addition, we observed that the kernels in the heavy fraction of the DropSort had higher sphericity compared to the kernels in the light fraction, while single kernels with high AF levels were more spherical (round) compared to kernels with low AF. Calibrating the DropSort prototype to sort out kernels with lower single kernel density and length and higher sphericity could further improve its sorting efficacy to reduce AF contamination in maize grain.

Size sorting was not successful in reducing AF levels in maize to acceptable levels. Both size and DropSorting were similar in reducing FUM contamination, but DropSorting took longer than size sorting. By removing small and broken kernels, it was possible to reduce FUM to below 4 ppm. Murphy, Rice, and Ross (1993) showed that broken kernels contained 10-fold higher FUM levels than intact kernels. Likewise, Sydenham, van der Westhuizen, Stockenstrom, Shephard, and Thiel (1994) reported that excluding fine particles reduced FUM levels by 26–69%. We thought that DropSorting could be confounded by the heterogeneity of kernel sizes, so we tested the effectiveness of combined size sorting and DropSorting in reducing mycotoxin contamination. This approach reduced the average FUM level to under 2 ppm but failed to reduce AF levels in highly contaminated samples from Tanzanian markets. Sorting was more effective for the Mississippi samples, which had lower AF levels. This is an indication that the degree of success in sorting grain based on physical kernel attributes could vary among grain lots with different toxicity levels.

Currently, the most common grain sorting approach for communities in low-resource environments involves manual visual sorting by removing discolored, insect damaged, broken kernels. This method may or may not help reduce toxin contamination; toxin reductions were seen in some studies (Afolabi et al., 2006; Kabak et al., 2006) and not in others; Mutiga et al. (2014) found that visual sorting was effective for reducing FUM but not for AF. The visual sorting in our study helped reduced FUM concentrations to 0.3 ppm in average in TZ18 maize samples. This demonstrates that kernel appearance is a good indicator of FUM contamination in maize kernels. We also found that healthy-looking kernels has significantly lower AF compared to unhealthy-looking kernels. This agrees with the study of Park (2002), in which sorting out physically damaged, discolored, shriveled, and odd-shaped kernels from the grain lot reduced AF levels by 40–80%. Likewise, Matumba, Van Poucke, Njumbe Ediage, Jacobs, and De Saeger (2015) reported that hand sorting left less than 6% of AF and less than 5% of FUM; they found that this method was much more efficient in the decontamination of maize grain compared to flotation/washing and dehulling methods. However, we found that some moderately defective maize kernels (those with slight shape deformation but showing no visible moldiness, breakage or discoloration) had also high AF levels. This illustrates that kernel deformation is also an indicator of AF contamination, which supports the inference that fungal colonization can influence the morphological development of maize kernels.

In this study, we found that some healthy-looking kernels had high AF concentrations. This shows that kernel appearance alone is not a perfect indicator of AF levels in grain. The difference in visual sorting effectiveness between AF and FUM contaminated grain is likely associated with the difference in the ways *A. flavus* and *F. verticillioides* colonize maize kernels. Previous studies reported that there are histological differences in the colonization processes of these two fungi (Shu et al., 2015, 2017), but the association between the fungal colonization biology and effectiveness of kernel sorting based on visual appearance needs further investigation.

Visual sorting under UV light (the BGYF test) was somewhat effective in detecting toxic kernels with high AF levels (>20 ppb). Non-BGYF kernels had on average 960-fold lower AF compared to that in BGYF kernels, but this test had a false negative rate of 28%. The BGYF test has been used to predict the presence of AF in maize (Bothast & Hesseltine, 1975; Maupin, Clements, & White, 2003; Shotwell & Hesseltine, 1981) and other food commodities such as cotton seed and pistachio nuts (Hadavi, 2005; Marsh et al., 1969). However, studies based on single kernels showed that BGYF is not reliable because it is associated with false positives and false negatives (Pearson, Wicklow, & Brabec, 2009, 2001; Wicklow, 1999; Yao et al., 2010). In our study, there were no false positives associated with the BGYF test, most likely because the kernels we used were sourced from ears inoculated with toxigenic *A. flavus*. In the case of natural infection by atoxigenic strains or in regions where non-toxigenic *A. flavus* strains have been used for biological control of AF in sub-Saharan Africa (Bandyopadhyay et al., 2016), the rate of false positives would presumably be much higher.

For AF-contaminated groundnut, visual sorting is the most widely used AF-mitigation method in low-resource environments. This method was effective in reducing AF in this study and in previous ones (Dickens and Whitaker, 1975; Chiou, Wu, & Yen, 1994; Filbert and Brown, 2012; Galvez, Francisco, Villarino, Lustre, & Resurreccion, 2003; Kaaya, Harris, & Eigel, 2006; Pelletier & Reizner, 1992, Xu et al., 2016). However, the levels of AF reduction vary among these studies. Visual sorting is time consuming and tedious for large grain lots if performed manually and is not affordable in low-resource environments if performed electronically by available optical sorters.

DropSorting and size sorting of groundnut kernels were based on HKW and not KBD, thus both of these sorting methods were targeting similar physical kernel attributes (mass and volume). Size sorting was more effective than DropSorting in grouping kernels based on AF concentrations. The difference in the sorting efficiency between size sorting and DropSorting could be due to the difference in rejection rates that were much higher for size sorting than for DropSorting. Therefore, excluding small and light groundnut kernels can reduce AF in groundnut grain. This agrees with the findings of Whitaker at al. (2005), who showed that higher AF levels were associated with smaller groundnut kernels. The association between small kernel size and AF contamination was due to the fact that immature groundnut pods (with smaller kernels) are more susceptible to *A. flavus* infection (Dorner, Cole, Sanders, & Blankenship, 1989). In the US groundnut industry, small kernels are usually used for oil production, therefore much of the AF ends up in the oil stock category (Dorner, 2008).

In summary, DropSort effectiveness in reducing mycotoxin levels varied depending on the type of toxin, the crop, and the levels of contamination. The DropSort grouped maize grain based on KBD and HKW. In most experiments, fractions with lower KBD and HKW had higher AF and FUM levels. The average FUM concentration was reduced to under the 4 ppm regulatory limit (Codex Alimentarius) after either DropSorting or size sorting. Size sorting followed by DropSorting was effective in reducing the average FUM level in maize kernels to below 2 ppm. In addition, visual sorting alone or in combination with DropSorting was also effective in reducing FUM to almost undetectable levels. Even though none of the sorting methods were effective in reducing AF levels in maize to below the U.S. FDA regulatory limit of 20 ppb, grain sorting was effective in stratifying kernels based on physical kernel features useful for more effective sampling for AF quantification. Sorting and reducing sampling variance may not be possible in the case of heavily AF contaminated grain. In addition, we identified bulk and single kernel attributes associated with AF levels. Single kernel attributes associated with AF levels may be useful to calibrate future devices like the DropSort for better sorting efficacy. For groundnut, both size sorting and DropSorting grouped kernels samples based on HKW. DropSorting did not significantly reduce AF concentrations, whereas size sorting and visual sorting were much more effective in separating groundnut grain based on AF levels. A low-cost sorter that involves different layers of sorting based on kernel physical and spectral features could be part of a successful strategy for managing AF contamination in food.

## Funding

This work was funded by the 10.13039/100000865Bill & Melinda Gates Foundation (OPP1155626). The research was supported in part by the U.S. Department of Agriculture, Agricultural Research Service. USDA is an equal opportunity provider and employer.

## Disclaimer

Mention of trade names or commercial products in this publication is solely for the purpose of providing specific information and does not imply recommendation or endorsement by the U.S. Department of Agriculture.

## CRediT authorship contribution statement

**Meriem Aoun:** Conceptualization, Methodology, Investigation, Formal analysis, Visualization, Writing - original draft. **William Stafstrom:** Methodology, Investigation. **Paige Priest:** Investigation. **John Fuchs:** Methodology. **Gary L. Windham:** Methodology, Investigation. **W. Paul Williams:** Resources, Methodology. **Rebecca J. Nelson:** Conceptualization, Methodology, Resources, Writing - review & editing.

## Declaration of competing interest

The authors declare no conflict of interest.
