# Supplementary material for: Low-cost grain sorting technologies to reduce mycotoxin contamination in maize and groundnut
Source: Food Control. 2020 Dec;118:107363. doi: 10.1016/j.foodcont.2020.107363 (PMC7439795; doi:10.1016/j.foodcont.2020.107363)
Supplement: Multimedia component 2 [file mmc2.docx]

**
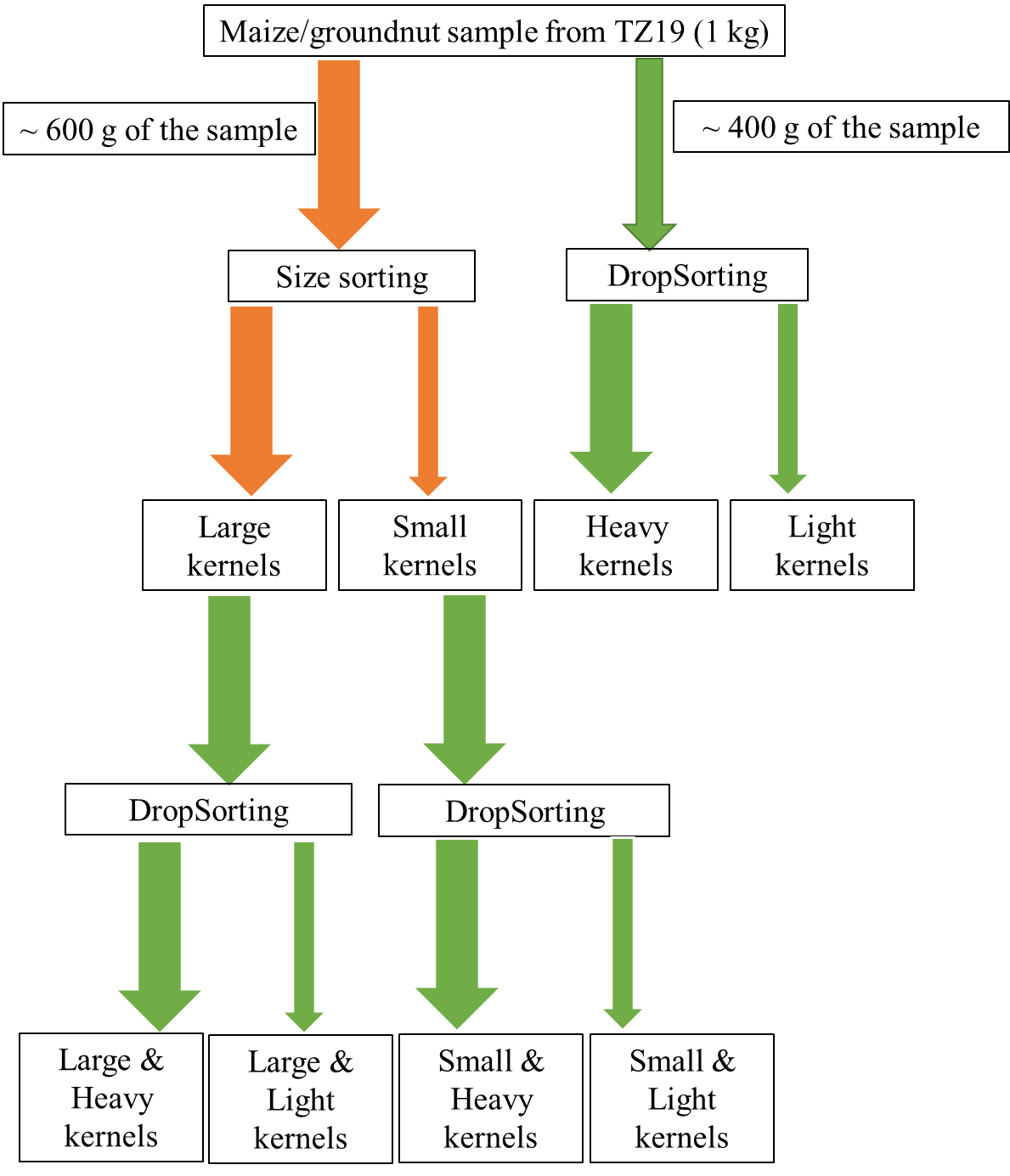
**

**Fig. S1. Grain sorting procedure of maize and groundnut samples from Tanzania in 2019.**

**
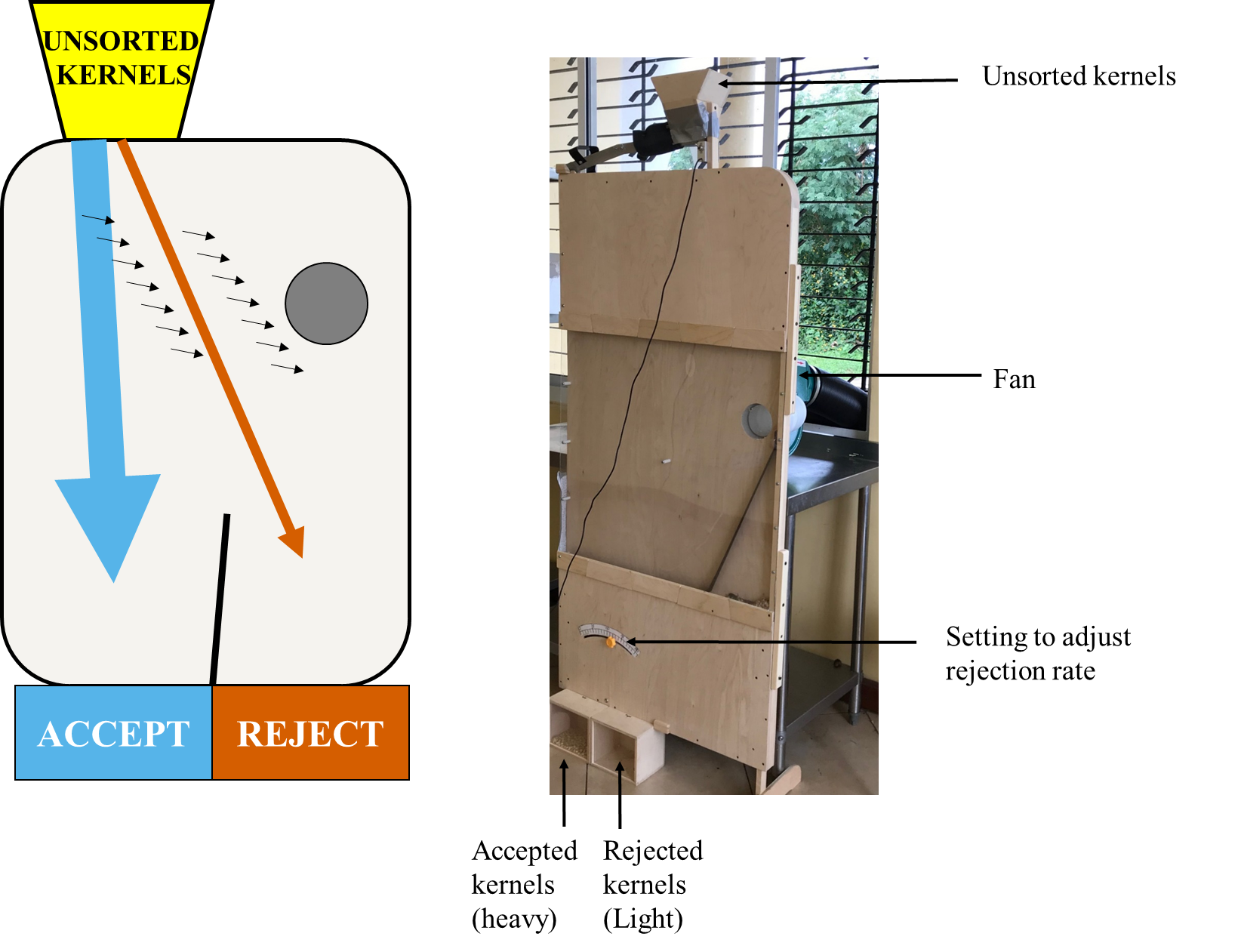
**

**Figure S2. DropSort prototype**

**
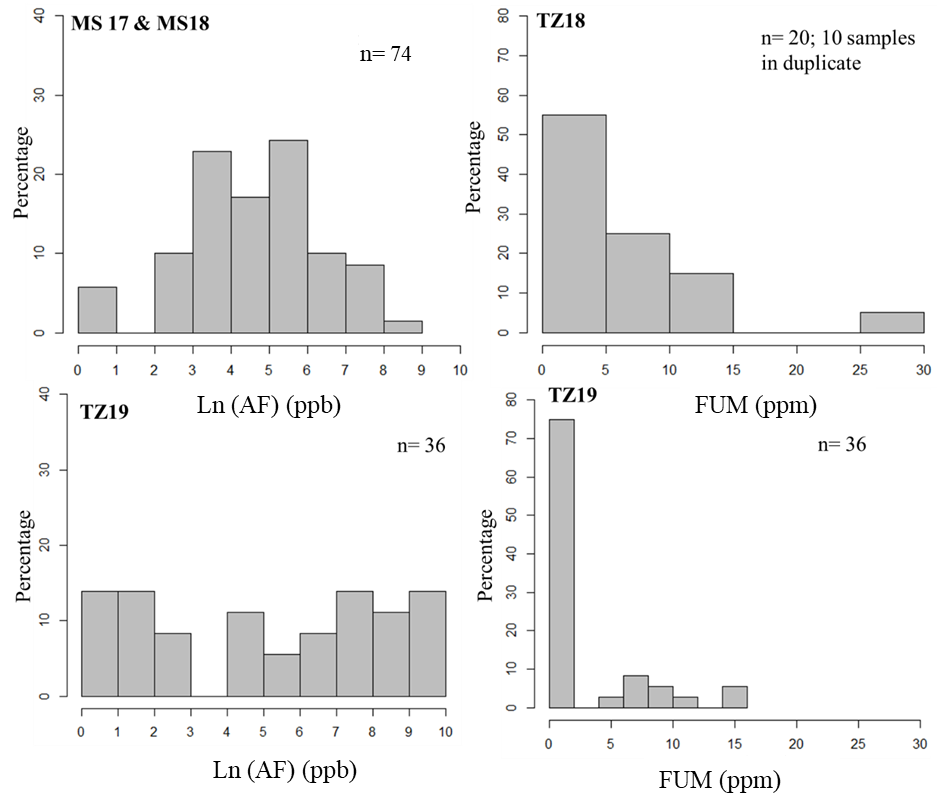
Fig. S3.** Distributions of log-transformed aflatoxins (AF) and fumonisins (FUM) in maize samples from experimental plots in Mississippi fields in 2017 (MS17) and 2018 (MS18) and in maize samples collected from open markets in Tanzania in 2018 (TZ18) and in 2019 (TZ19).


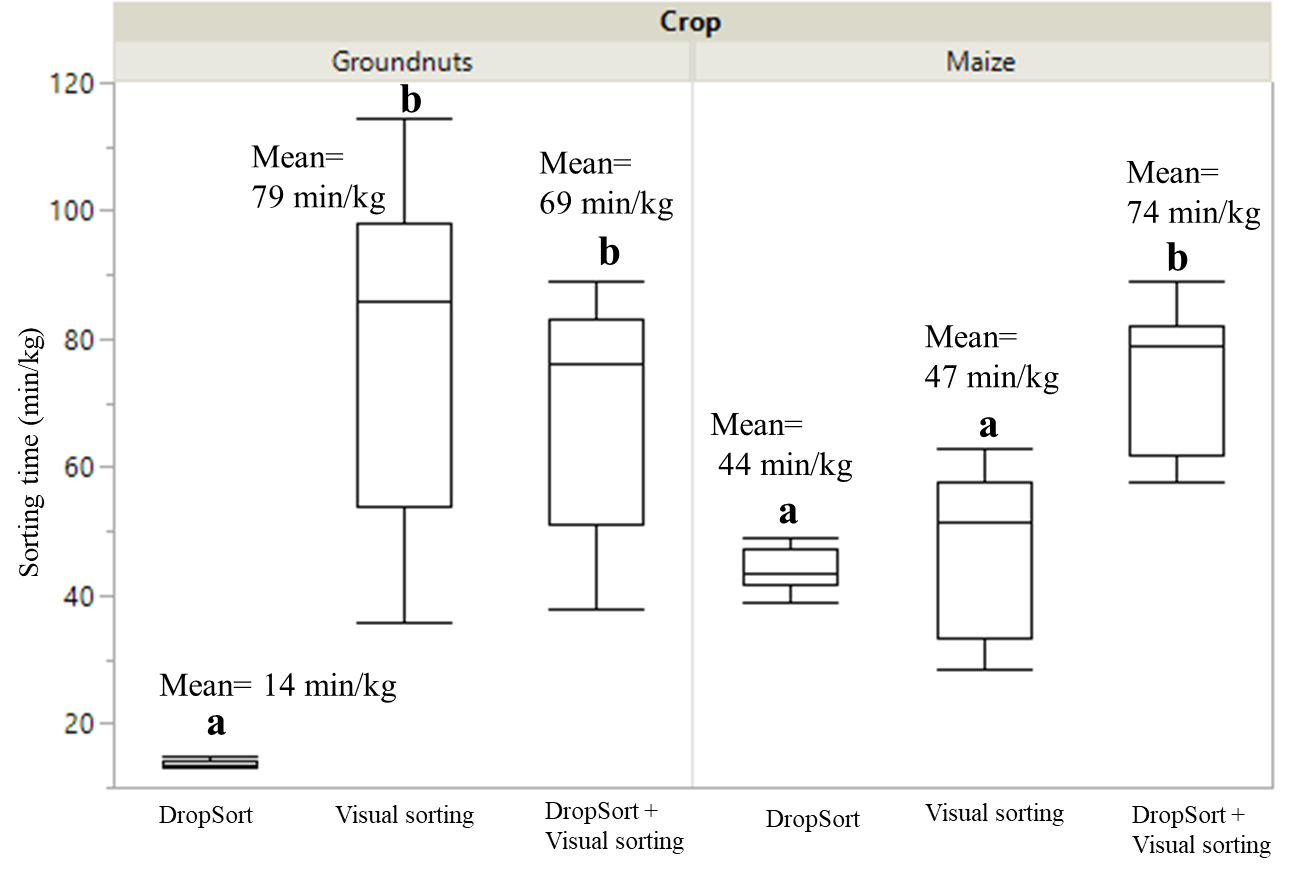


**Figure S4.** Sorting time efficiency of DropSorting, visual sorting, and DropSorting followed by visual sorting of maize and groundnut grain. The letters on the boxplots represent results of Tukey’s test (treatments with different letters are significantly different at 95% level of confidence).

**
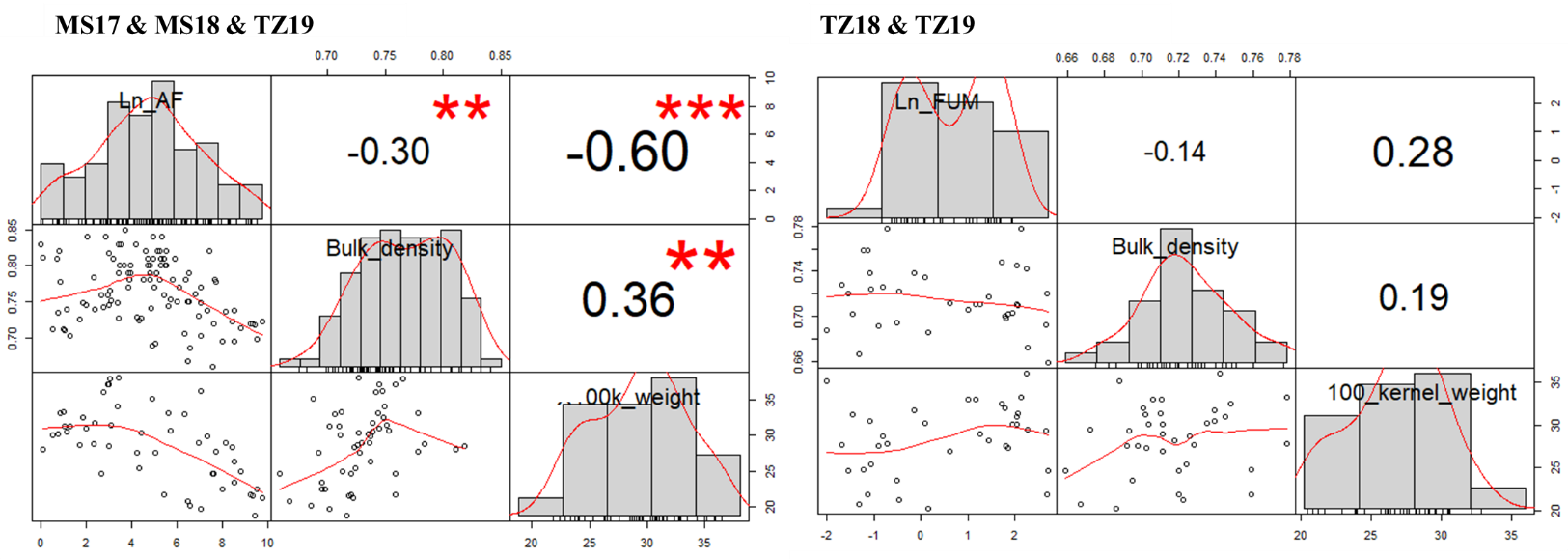
**

**Fig. S5.** Correlations between kernel bulk density and 100 kernel weight and mycotoxin concentrations in unsorted maize samples from Mississippi (MS17 and MS18) and Tanzania (TZ18 and TZ19). The stars represent the significance levels of the correlation. ** and *** corresponds to significant correlation at 99% and 99.9% level of confidence, respectively.


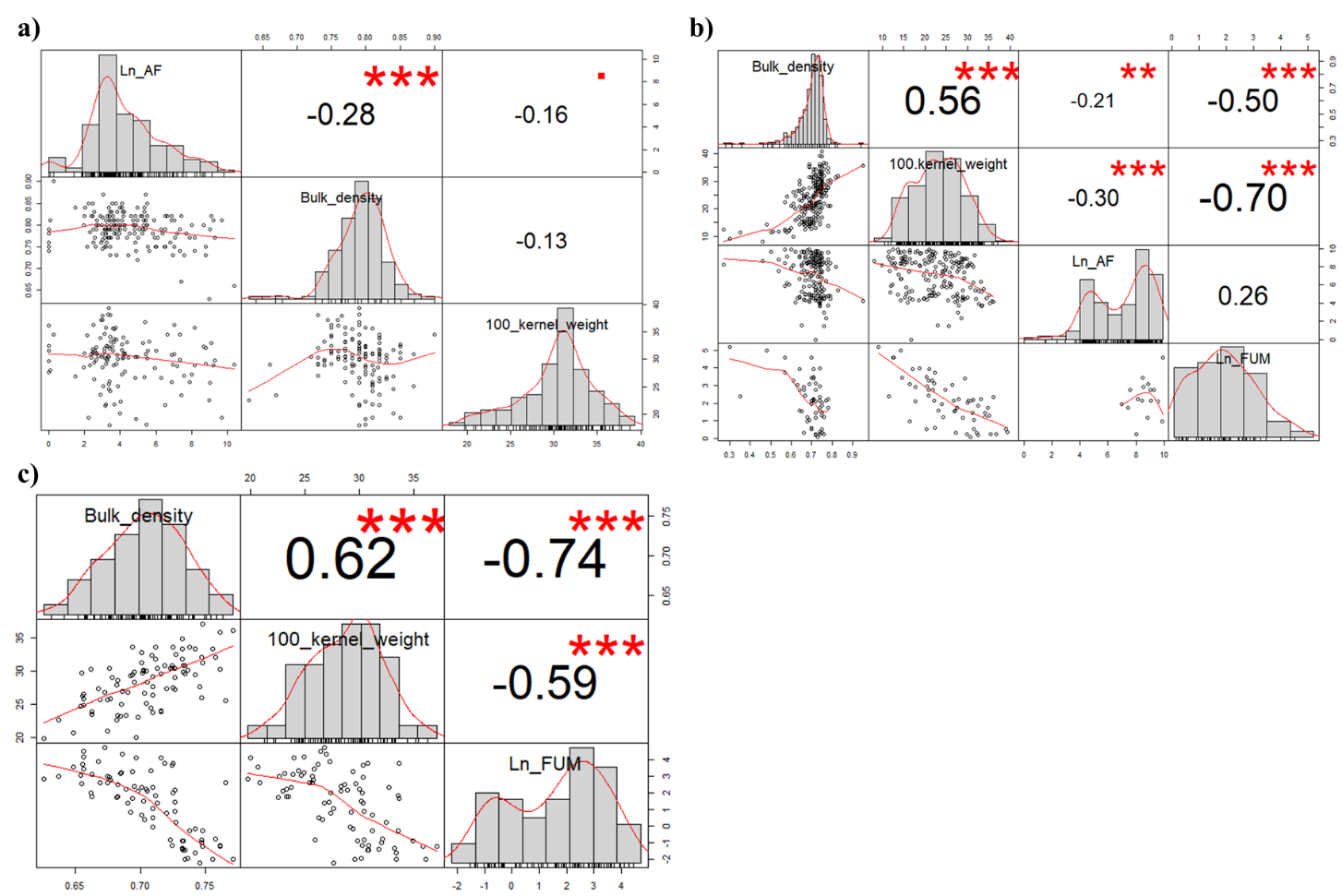


**Fig. S6**. Bulk kernel attributes of maize samples associated with aflatoxin and fumonisin levels in combined unsorted and sorted maize samples. a) Samples from US inoculated maize with *A. flavus* in MS17 & MS18, b) naturally infected maize samples from Tanzania in 2019, and c) naturally infected maize samples from Tanzania in 2018. The small red square, **, and *** corresponds to significant correlation at 90%, 99%, and 99.9% level of confidence, respectively.

**
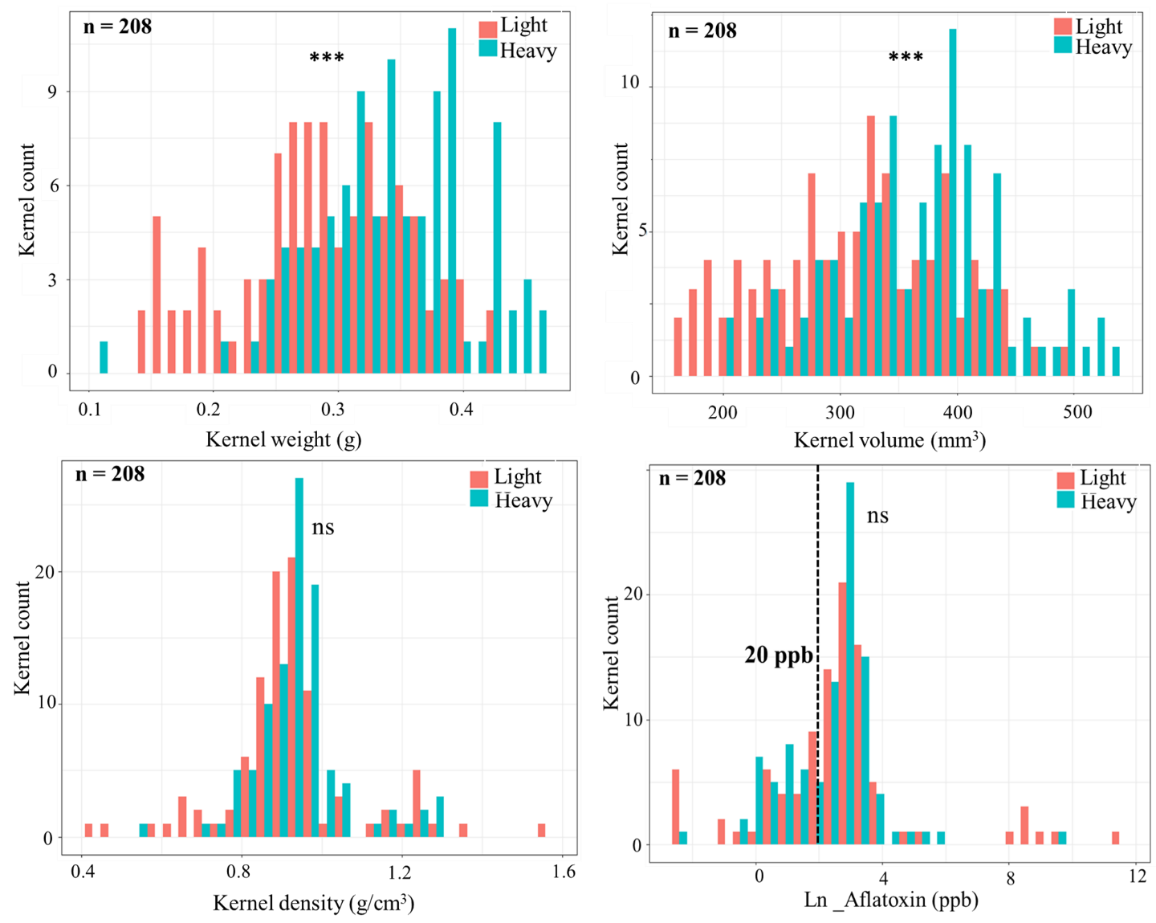
**

**
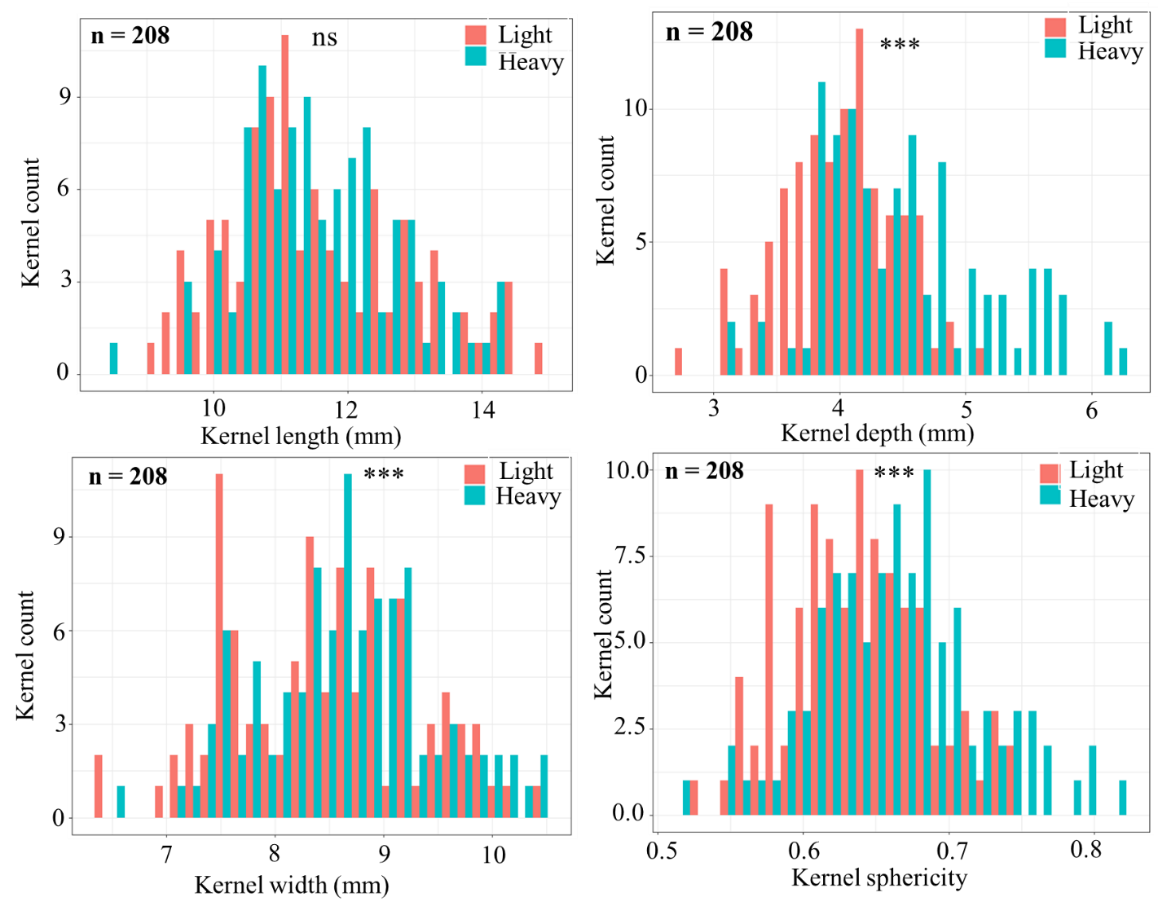
**

**Fig. S7**. Distribution of diverse measures of single kernels in the heavy and light fractions of the DropSort. Comparisons were based on two-tail t test (treatments with *** are significantly different at 99.9% level of confidence, ns: no significant difference at 95% level of confidence).

**
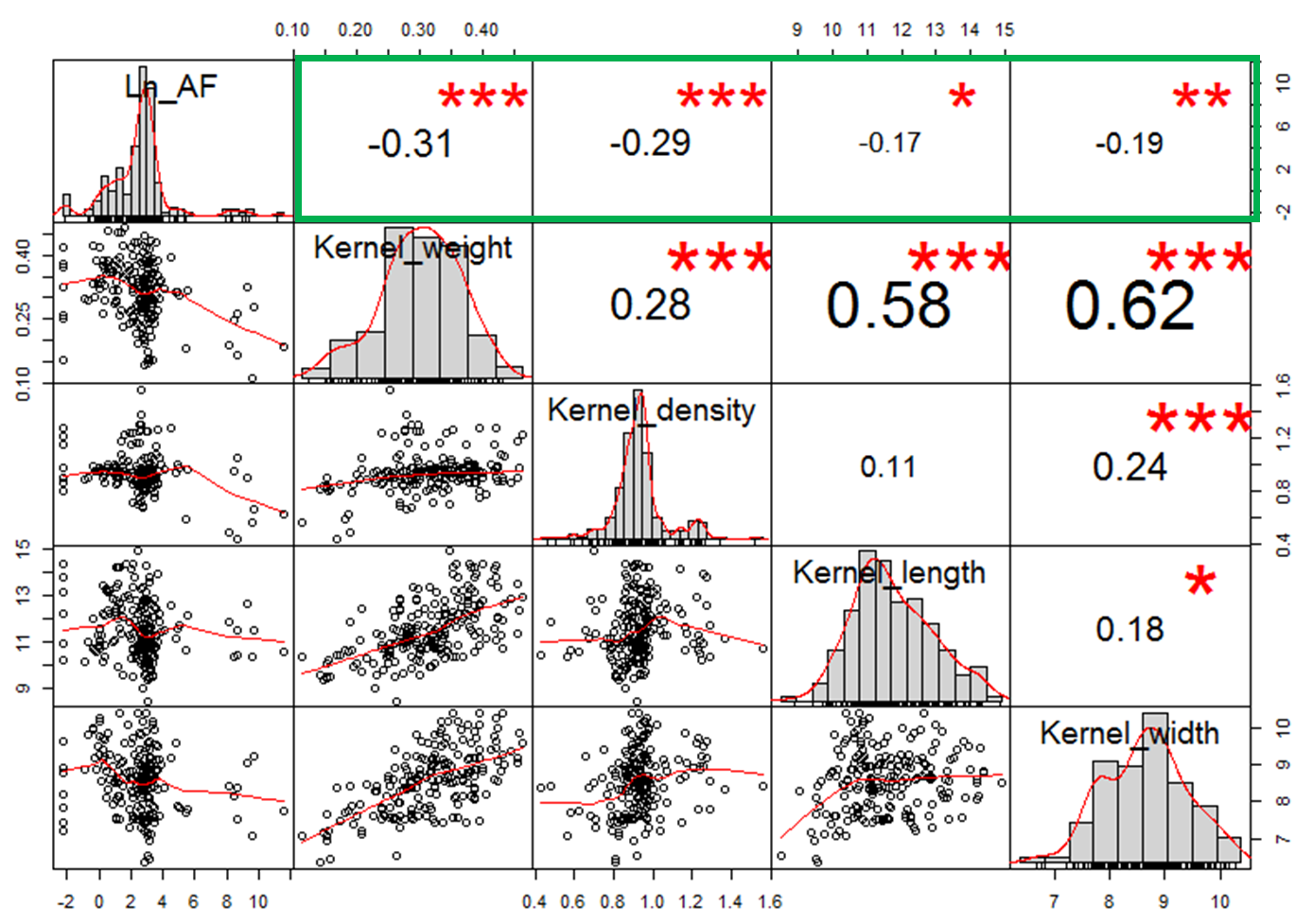
**

**Fig. S8.** Correlations between physical kernel attributes and aflatoxin levels in single maize kernels. The kernels were from nine hybrids grown and inoculated with *A. flavus* in Mississippi in 2017. *, **, and *** corresponds to significant correlation at 95%, 99%, and 99.9% level of confidence, respectively.


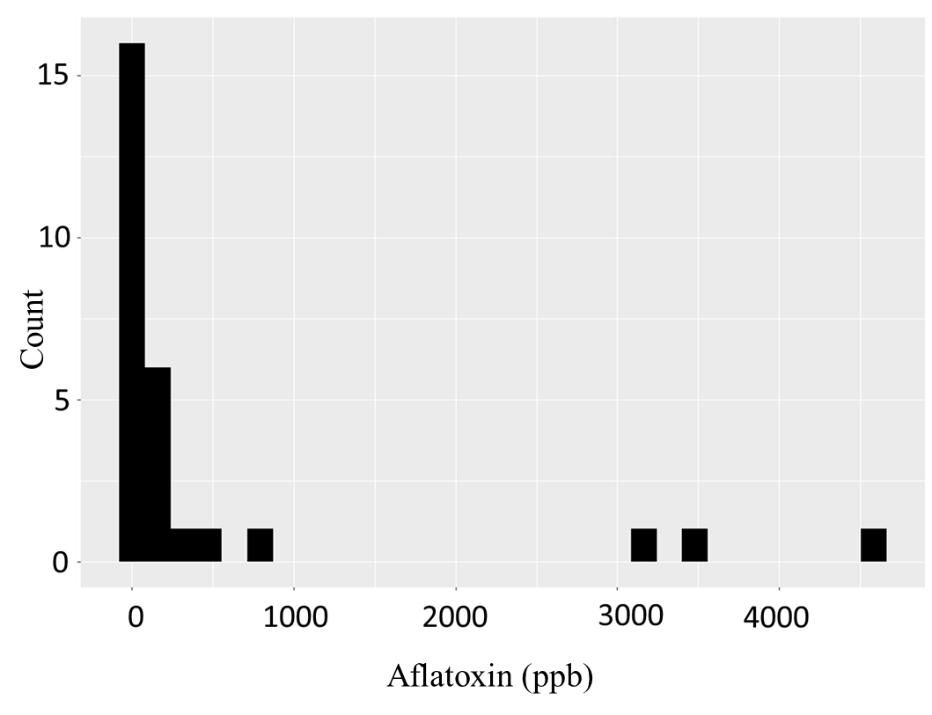


**Fig. S9**. Distributions of aflatoxins in naturally infected groundnut samples collected from Tanzania in 2019.

**
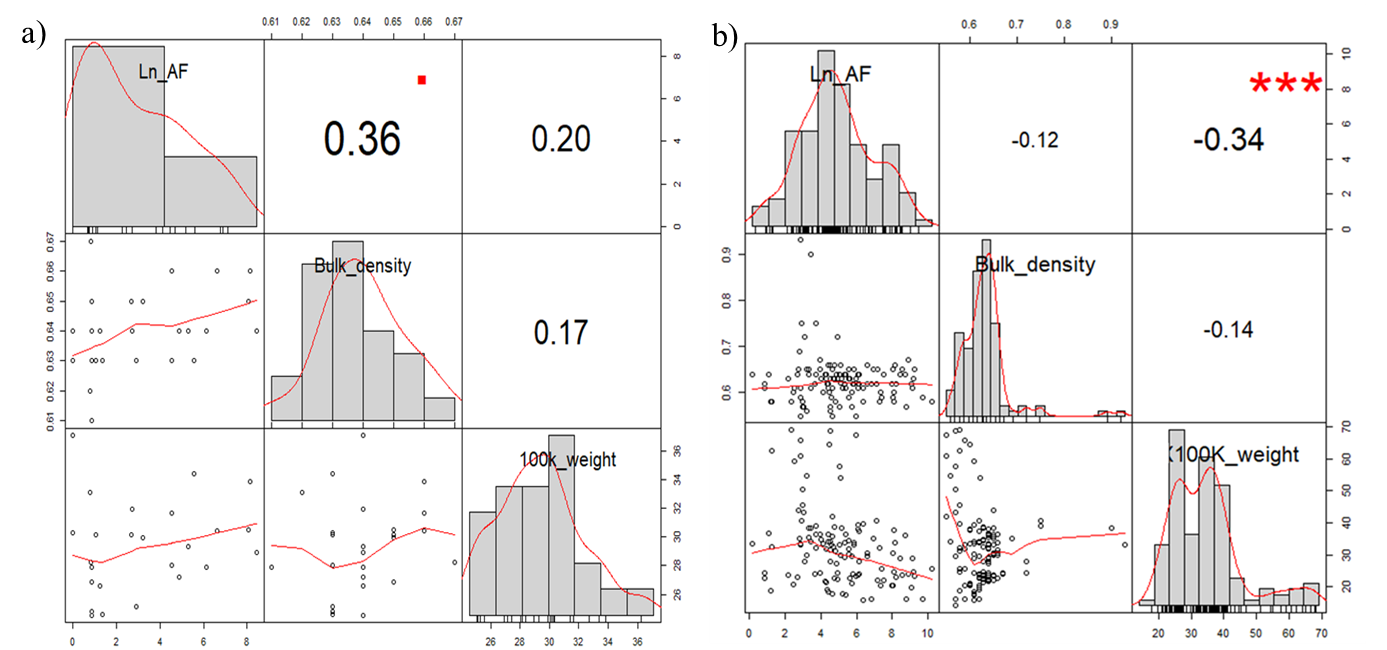
**

**Fig. S10**. Correlations between bulk kernel attributes (bulk density and 100 kernel weight) and aflatoxin concentrations in groundnut samples from Tanzania in the year of 2019. a) Correlations in unsorted groundnut samples (n=28) and b) correlations in combined sorted and unsorted groundnut samples. The small red square and *** corresponds to significant correlation at 90% and 99.9%, respectively.
